# They approve but they don’t act: promoting sustainable minority behavior with (conflicting) social norm appeals

**DOI:** 10.3389/fpsyg.2024.1337585

**Published:** 2024-06-26

**Authors:** Anna Schorn, Werner Wirth

**Affiliations:** Department of Communication and Media Research, University of Zurich, Zürich, Switzerland

**Keywords:** social norms, social norm appeal, explainer video, persuasion, experiment, nudging, social influence, sustainability

## Abstract

**Background:**

Social norm appeals are effective in promoting sustainable majority behavior but could backfire when the target behavior is only performed by a minority of people. However, emphasizing that an increasing number of people have started engaging in the behavior or that the majority approve the behavior might prevent such negative effects. However, only a few studies have investigated the combination of descriptive minority and injunctive majority social norm appeals, with inconsistent results. Some studies of minority behavior suggest that the characteristics of recipients might determine the inconsistent results regarding the impact of minority social norm appeals and that social norm appeals could have a greater impact on individuals with weaker environment related dispositions.

**Method:**

Two two-wave studies investigated how descriptive minority appeals, injunctive majority appeals, and their combination can be used to motivate sustainable minority behavior and what role environment related dispositions play in this context. In the first part, perceived social norms, environment related dispositions, behavioral intentions, and pre-attitudes were measured. Two weeks later, respondents participated in a 3 (descriptive social norm appeal: static vs. dynamic vs. absent) × 2 (injunctive majority social norm appeal: present vs. absent) between-subjects experiment and watched an explainer video on voluntary carbon offsets that included various social norm appeals.

**Results:**

In both studies, we found that social norm appeals influenced perceived social norms. However, persuasive effects were only observed in the first study, and an injunctive majority appeal increased persuasive outcomes, but there were no differences between the descriptive conditions and no differences in their impact depending on individuals’ environment related dispositions in either study.

**Discussion:**

A single exposure may be insufficient to exert persuasive effects and the change in perceived social norms may first need to be internalized. In online explainer videos, the effects of social norm appeals could be amplified by algorithm-based suggestions and when social norm appeals draw attention to norm-conforming content. However, further research is required on the long-term effects and their possible amplification via social media.

## 1 Introduction

Individuals and households are responsible for a considerable proportion of global emissions. Therefore, behavior change is needed on a societal and individual level, and people must be motivated to change their behavior to mitigate climate change (Hertwich and Peters, 2009; Fell and Traber, 2020; IPCC, 2021). In recent years, this issue has gained increasing attention in politics and the mass media, and various studies have shown that a high level of awareness of climate change exists in large parts of the world (e.g., Lee et al., 2015; Oliver and Adkins, 2020; Baiardi and Morana, 2021; Boon-Falleur et al., 2022; Carmichael et al., 2023; Andre et al., 2024). However, only a few people seem to be actively tackling the related problems by changing their behavior (Brechin and Bhandari, 2011; European Commission, 2020, 2021; Passafaro, 2020; Andre et al., 2024). Therefore, it remains a challenge for scientists and practitioners to determine how individuals can be motivated to adopt sustainable behaviors.

One of the most important interventions in behavioral science to change behavior in general and motivate sustainable behavior in particular is the use of social norm appeals (Cialdini and Jacobson, 2021). This is a simple, inexpensive, and effective method of achieving compliance by changing the perceived prevalence of a particular behavior and its approval, usually by highlighting that the desired behavior is prevalent or has gained wide approval in certain social contexts (Mortensen et al., 2019; Rhodes et al., 2020). When using social norm appeals, individuals are informed about the proportion either of those who engage in the target behavior (descriptive social norm appeal) or of those who approve of the target behavior (injunctive social norm appeal), both within a reference group (Cialdini et al., 1990, 1991).

Descriptive and injunctive social norm appeals are successful and proven interventions when the target behavior is performed and approved by the majority (e.g., Rhodes et al., 2020). However, if the target behavior is not widely adopted (descriptive minority), social norm appeals risk undesirable backfire effects when people learn that their unsustainable behavior is the norm (e.g., Schultz et al., 2007; Richter et al., 2018). In this case, normative information can have the opposite effect of what the communicator intends (e.g., Cialdini, 2003; Schultz et al., 2007; Richter et al., 2018; Berger, 2021).

Such backfire effects of descriptive minority appeals can be prevented, on the one hand, by not only highlighting the minority of people performing the target behavior (static descriptive appeal), but by presenting the behavior as a growing trend (dynamic descriptive appeal) that people increasingly follow (Sparkman and Walton, 2017). Most studies examining dynamic appeals have revealed positive effects when people anticipate ongoing change and a future world in which that minority behavior is the norm (e.g., Sparkman and Walton, 2017; Loschelder et al., 2019; Mortensen et al., 2019). On the other hand, injunctive majority appeals can be used to indicate the proportion of people who approve of the behavior rather than the minority of people who perform the target behavior (Schultz et al., 2007; Rhodes et al., 2020). However, the positive effects may be attenuated or even reversed when an injunctive majority appeal is combined with a descriptive minority appeal or when it is obvious that the target behavior is only performed by a minority.

Research on social norm conflict shows that social norm appeals can be ineffective when injunctive majority appeals do not match descriptive minority appeals or salient descriptive norms (e.g., Schultz et al., 2008; Smith et al., 2012). People may experience an internal conflict or cognitive dissonance that could suppress the desired behavior when they experience that the usual behavior does not correspond to what should be done (cf. Thøgersen, 2008; Jacobson et al., 2020). However, recent studies of environmental communication (e.g., Schorn and Wirth, 2023) and health communication (e.g., Habib et al., 2021) have found no evidence of social norm conflict. It is therefore unclear under which circumstances conflicting social norm appeals can promote or suppress sustainable behavior. However, various studies of social norm appeals suggest that there may be differences in effects depending on the characteristics of the sample. Studies applying social norm appeals have shown that they seem to have a stronger effect on individuals with weaker environmental attitudes, while individuals with stronger environmental attitudes seem less affected (e.g., de Groot et al., 2021; Aruta, 2022). Accordingly, backfire effects seem more likely among people with weak pro-environmental attitudes compared with those with strong environmental attitudes (Richter et al., 2018). However, these results are often implicit or retrospective and do not control for environment related dispositions (Demarque et al., 2015; Richter et al., 2018; Aruta, 2022), and there remains a lack of research that explicitly addresses the effects of sample characteristics in the context of social norm interventions to test these assumptions.

Therefore, this study aimed to investigate how descriptive and injunctive social norm appeals, and their combination can be used effectively to motivate sustainable minority behavior, and the role of environment related dispositions in this context. Because most studies focusing on social norm conflict have not included dynamic descriptive appeals (e.g., Smith et al., 2012) and studies on dynamic descriptive appeals have not addressed social norm conflict (e.g., Sparkman and Walton, 2017), we examined if a static descriptive minority appeal combined with an injunctive majority appeal results in negative effects, and if these can be prevented by using a dynamic descriptive appeal instead. We report on two two-wave studies that allowed us to measure environment related dispositions and perceived social norms independently of social norm appeals. In this way, this study aims to contribute to basic research on social norm appeals that promote environmental behavior approved by a majority but performed by only a minority. In the two experiments, different social norm appeals were embedded in explainer videos on voluntary carbon offsets. Explainer videos are very popular on social media and are thought to be more effective than written text in changing perceptions of social norms and the resulting beliefs or behaviors (e.g., Rhodes et al., 2020). Thus, our research extends existing research by combining injunctive appeals with dynamic descriptive appeals and examining the role of environment related dispositions in relation to conflicting social norm appeals, measuring them separately in time from the manipulation.

## 2 Theoretical background: social norm appeals

The focus theory of normative conduct posits that social norms powerfully and systematically influence human behavior (Cialdini et al., 1990, 1991). According to this theory, there are descriptive social norms that reflect people’s typical behavior, and injunctive social norms that indicate what behavior is desirable or approved. Social norms can be activated or made salient so that they can guide behavioral decisions. Accordingly, social norm appeals attempt to change behavior by modifying the prevailing view that a particular behavior is more prevalent or has gained wide approval in a certain social context (Mortensen et al., 2019; Rhodes et al., 2020; Cialdini and Jacobson, 2021). Social norm appeals can change perceptions and increase the salience of existing norms, which can lead to conformity (Miller and Prentice, 1996; Abrahamse and Steg, 2013; Farrow et al., 2017; Poškus, 2018; Rhodes et al., 2020). Descriptive social norm appeals provide information about the proportion of people who engage in the desired behavior, and injunctive social norm appeals describe the proportion of people approving the behavior, both within a reference group (Cialdini et al., 1991; Schultz et al., 2007; Goldstein et al., 2008). Descriptive social norms can influence behavior based on social proof, as they indicate behavior that has proven to be effective for others (Jacobson et al., 2011). Injunctive social norms can influence behavior by creating social pressure to conform because they show what behavior a social group approves or expects. Therefore, descriptive norm appeals work heuristically and nonconsciously through the peripheral route of information, while injunctive appeals need greater elaboration to consciously make the “right” choice, particularly when other people do not act accordingly (cf. Göckeritz et al., 2009; Melnyk et al., 2019).

### 2.1 Descriptive social norm appeals

Descriptive social norm appeals can lead to compliance when targeting majority behavior because they work as social proof, showing which behavior has proven to be effective for other people (Jacobson et al., 2011). However, descriptive appeals are ineffective and run the risk of undesirable boomerang or backfire effects when the target behavior is not prevalent and people learn that their unsustainable behavior is actually the norm (Reno et al., 1993; Schultz et al., 2007; Smith and Louis, 2009; Elgaaied-Gambier et al., 2018; Loschelder et al., 2019; Berger, 2021). Most studies on descriptive minority appeals conclude that they are not effective to promote sustainable behavior (e.g., Richter et al., 2018; Shealy et al., 2018; Aruta, 2022). Highlighting the minority behaving in the desired way does not act as social proof and therefore can result in a contrary behavioral adjustment. In this case, a descriptive minority appeal performs worse than a message without any normative information. Thus, normative information can backfire when people follow the majority not acting sustainable. Therefore, we hypothesize that a message containing a static descriptive minority appeal will weaken the perceived prevalence of the behavior and be less effective in promoting sustainable behavior than a message without a descriptive appeal (backfire effect).

*H1*: A message including a static descriptive minority appeal (a) negatively affects the perceived prevalence of the behavior and (b) is less persuasive than a message without a descriptive minority appeal.

Nevertheless, backfire effects of static descriptive minority appeals can be prevented by presenting the behavior as a trend that people increasingly follow. Such dynamic descriptive appeals can lead to preconformity and compliance when individuals anticipate ongoing change and a future world in which that minority behavior is the norm (Sparkman and Walton, 2017). In this case, individuals know that the behavior is currently being performed by a numerical minority of people (perceived descriptive minority), but it is anticipated that the behavior could be performed by a majority in the future (increasing future descriptive norm). Several studies have shown that dynamic descriptive appeals hold promise for promoting sustainable minority behavior (e.g., Sparkman and Walton, 2017; Loschelder et al., 2019; Mortensen et al., 2019). However, recent studies suggest that dynamic appeals are more likely to catch backfire effects from static minority appeals, as the overall results are weaker when they were compared against control groups without social norm appeals (e.g., DellaValle and Zubaryeva, 2019; Carfora et al., 2022; Gossen et al., 2023). Therefore, we hypothesize that a dynamic appeal increases the perception of a trend and is more effective in promoting sustainable minority behavior than a static minority appeal, replicating results of former studies.

*H2*: A message including a dynamic descriptive norm appeal (a) leads to a stronger perception of a trend and (b) is more persuasive than a message including a static descriptive minority appeal.

Loschelder et al. (2019), Mortensen et al. (2019), Schorn and Wirth (2023), and Sparkman and Walton (2017) suggest that dynamic appeals work by reinforcing the perception of a trend, which can lead to preconformity. Following this reasoning, a dynamic appeal should also lead to an increased perception of a trend and be more effective than messages without a descriptive appeal, despite the mixed results of other studies.

*H3*: A message including a dynamic descriptive norm appeal (a) leads to a stronger perception of a trend and (b) is more persuasive than a message without a descriptive appeal.

### 2.2 Injunctive social norm appeals

Another strategy for preventing the negative effects of static descriptive minority appeals is to emphasize that an injunctive majority approves of the behavior, rather than referring to a descriptive minority performing the behavior. People appear to systematically underestimate the approval of various environmental behaviors in the population (Nolan, 2021; Wolf et al., 2023; Andre et al., 2024). Injunctive social norm appeals can adjust such misperceptions and increase perceived injunctive norms. However, few studies have used injunctive appeals that address minority behavior or vary the strength of the appeal (e.g., Schultz et al., 2008; de Groot and Schuitema, 2012; Smith et al., 2012; Schorn and Wirth, 2023). In addition, injunctive appeals are often operationalized differently, which makes it difficult to compare and classify the results (Smith and Louis, 2009; Shulman et al., 2017; Schorn et al., 2023). For example, in studies using personalized normative feedback, the manipulation of injunctive norms has commonly been indirect, using smileys or other icons to indicate whether the behavior was within a desired range (e.g., Schultz et al., 2007; Bergquist and Nilsson, 2016; Bhanot, 2018; for review Bergquist et al., 2019). Such injunctive messages serve as a signal of how the measured behavior has been evaluated. These injunctive messages seem to be effective to prevent backfire effects in the context of normative feedback. However, since the injunctive norm (share of people approving the behavior) is not described directly within a reference group, they should not operate via a change in perceived injunctive social norms. Injunctive icons indicate desirable behavior (e.g., for scientific, economic or sustainability reasons), but not necessarily socially desirable behavior. Therefore, the results of studies on normative feedback cannot be directly transferred to research on social norm appeals.

Nevertheless, injunctive appeals seem to be well suited to promote pro-environmental behavior (Rhodes et al., 2020) and increase public support for climate policy measures (Nolan, 2021) and the acceptance of a behavior (de Groot and Schuitema, 2012; Schorn and Wirth, 2023). Although there are only a few studies examining injunctive appeals in the context of minority behavior (e.g., Smith et al., 2012; Schorn and Wirth, 2023), yielding partly contradictory results, we assume that injunctive majority appeals should have a positive effect overall. We hypothesize that a message containing an injunctive majority appeal will increase the perceived prevalence of approval and be more effective in promoting sustainable minority behavior than a message without an injunctive appeal.

*H4*: A message including an injunctive majority appeal (a) increases the perceived prevalence of approval and (b) is more persuasive than a message without an injunctive majority appeal.

However, due to methodological differences, unanswered questions remain about the effectiveness of an injunctive appeal stating majority approval in the context of minority behavior. Studies of conflicting social norms have yielded inconsistent results regarding their effects, and it remains unclear how conflicting descriptive and injunctive social norm appeals influence each other (cf. Smith et al., 2012; Habib et al., 2021).

### 2.3 Conflicting social norms

Studies demonstrating the effectiveness of injunctive social norm appeals often work with majority approval and contrast them with descriptive majority appeals (e.g., Rhodes et al., 2020). In the context of sustainable behavior, individuals indeed often have prevalent positive attitudes and seem to approve of sustainable behavior in general and of specific actions, but they have not yet adapted their own behavior to the same extent (European Commission, 2020; Baiardi and Morana, 2021; Economist Intelligence Unit, 2021). Thus, the initial situation for new sustainable behaviors commonly includes an injunctive majority and a collective descriptive minority social norm (actual prevalence of the behavior, cf. Chung and Rimal, 2016). This can be problematic because injunctive and descriptive social norms influence each other, particularly when they are not aligned or congruent (Eriksson et al., 2015; Cialdini and Jacobson, 2021).

Individuals infer social norms through their observation of others, personal and media communication, and self-knowledge (Cialdini et al., 1991; Miller and Prentice, 1996; Witzling et al., 2019). Therefore, the effect of an injunctive majority appeal can be influenced by a descriptive appeal and the fact that people have an idea of the frequency of occurrence of a behavior even without a descriptive minority appeal. Conversely, an injunctive majority appeal can increase not only perceived injunctive but also perceived descriptive social norms (Eriksson et al., 2015). When an injunctive majority is emphasized, this may have spillover effects on perceived descriptive norms, and individuals may overestimate the frequency of occurrence (Eriksson et al., 2015; Rhodes et al., 2020). In this case, the mention of the injunctive majority serves as an anchor for estimating descriptive norms (Tversky and Kahneman, 1974). However, it is unclear how descriptive and injunctive appeals influence each other and how their interaction impacts perceived social norms (Cialdini and Jacobson, 2021). Accordingly, we address the question of how the combination of descriptive and injunctive appeal influences perceived social norms.

RQ1: How do conflicting social norm appeals influence perceived social norms?

Regarding the persuasion effect, research on social norm conflict (Smith et al., 2012) or the norm alignment effect (Bergquist and Nilsson, 2016) suggests that social norm appeals may be ineffective when the injunctive (majority) appeals do not match the salient descriptive (minority) norms, and vice versa (e.g., Schultz et al., 2008; Smith and Louis, 2008; Smith et al., 2012; McDonald et al., 2014b; Bonan et al., 2020; Ge et al., 2020; Jacobson et al., 2020). When individuals learn that the common behavior does not reflect what should be done, they can experience an inner conflict or cognitive dissonance, which can suppress the desired behavior (cf. Schultz et al., 2008; Thøgersen, 2008; Smith et al., 2012; McDonald et al., 2014a; Jacobson et al., 2020).

Within the context of personalized normative feedback, the alignment of descriptive and injunctive feedback to prevent backfire effects coming along with descriptive minority norms has frequently been confirmed, referred to as norm alignment effect (e.g., Schultz et al., 2007; Allcott, 2011; Bergquist and Nilsson, 2016; Schultz et al., 2016; Bhanot, 2018; Bonan et al., 2020). In this scenario, injunctive icons (e.g., smileys) have been used to provide positive feedback: The individual behavior was not the norm (e.g., average neighbors perform worse), but it was supported and considered exemplary. Nevertheless, a recent conceptual replication study by Alblas et al. (2023) suggests that effects of normative feedback might be caused by regression to the mean rather than the manipulation of descriptive social norms which is why they did not find effects of the combination of descriptive and injunctive normative feedback.

When considering persuasive effects of conflicting social norm appeals, research shows as well that the alignment of social norm appeals could be decisive for their effects. For example, Schultz et al. (2008) combined an injunctive majority (“many”) versus a minority (“some”) appeal with a descriptive appeal and determined whether a majority (75%) versus a minority (25%) reused their towels. They showed a significant difference between the aligned majority social norm appeals and all other conditions, with the aligned majority social norm appeals being the most effective. However, they reported only the results for this contrast. Moreover, Smith et al. (2012) did not find main effects for descriptive minority versus majority appeals and injunctive appeals, but they found an interaction: the intention to conserve energy was lower when a descriptive majority appeal was complemented with an injunctive minority appeal or vice versa than when combining a descriptive majority appeal with an injunctive majority appeal. However, when using an injunctive minority appeal, there were no significant differences between majority and descriptive minority appeals. Therefore, participants receiving aligned majority appeals reported stronger intentions than participants in either the unaligned conditions or the aligned minority conditions.

However, Habib et al. (2021) found positive effects of conflicting social norm appeals and reported that the combination of an injunctive majority appeal and a static descriptive minority appeal increased organ donation. Furthermore, only one study has investigated the combination of injunctive majority appeals and dynamic descriptive minority appeals, finding no interactions between an injunctive majority appeal and different descriptive minority appeals (Schorn and Wirth, 2023). Nevertheless, most studies focusing on social norm conflict have not included dynamic descriptive appeals (e.g., Smith et al., 2012; Ge et al., 2020; Habib et al., 2021), whereas studies examining dynamic descriptive appeals have not addressed social norm conflict (e.g., Sparkman and Walton, 2017; Mortensen et al., 2019). Therefore, further research is needed to determine whether there is a conflict between static descriptive minority appeals and injunctive majority appeals and how potential negative effects could be mitigated by supplementing an injunctive majority appeal with a dynamic descriptive minority appeal. To address this question, we investigate if a message containing an injunctive majority appeal will be more effective in promoting sustainable behavior when combined with a dynamic descriptive appeal, a static descriptive appeal or a message without any descriptive appeal.

RQ2: How do conflicting social norm appeals influence persuasive outcomes?

### 2.4 Social norm appeals and environment related dispositions

One reason for the contradictory results with regard to social norm conflict could be individual’s environment related dispositions. Several studies have indicated that sample characteristics may influence the effects of social norm appeals (e.g., Demarque et al., 2015; Schultz et al., 2016; Lalot et al., 2018; Richter et al., 2018; de Groot et al., 2021; Aruta, 2022; Carfora et al., 2022). For example, Aruta, (2022) investigated gender differences and descriptive norm appeals, as women seem generally to have stronger pro-environmental attitudes than men. Men in the descriptive majority condition reported a higher intention to reduce their plastic consumption than did men in the minority condition, while there were no differences for women. De Groot et al. (2021) examined the interactions between social norm appeals and personal norms. Personal norms guide behavior through the perception of how a person would (dis)approve of his or her own behavior (Cialdini et al., 1991). For participants with weak personal norms regarding meat consumption, a descriptive majority appeal resulted in stronger intentions to reduce meat consumption than a descriptive minority appeal (de Groot et al., 2021). For medium and strong personal norms, there were no differences based on the type of the descriptive appeal. Similarly, Carfora et al. (2022) investigated the moderating role of intrinsic motivation, which they operationalized like personal norms, concluding that a dynamic descriptive appeal seems to be particularly effective among people with relatively weak intrinsic motivation.

Overall, descriptive norm appeals appear to be more effective in individuals with weak environmental attitudes or in populations that are generally less inclined to engage in sustainable behaviors. However, there are no studies that have examined social norm appeals in combination with or controlling for general characteristics of the sample, such as environmental awareness. Moreover, the above studies included only descriptive but not injunctive appeals or their combination. We hypothesize that the effect of different social norm appeals would generally be stronger in people with weak pro-environmental dispositions than in those with strong pro-environmental dispositions.

*H5*: The effects of different social norm appeals are more pronounced in people with weaker environment related dispositions than in those with strong ones.

### 2.5 The present research

To test these assumptions and answer the research questions, we conducted two experiments built on each other, which resulted from a series of contiguous studies (Schorn et al., 2023). Both experiments were two-wave studies to obviate the problem of measuring moderators or covariates before the stimulus, which may induce priming effects, whereas a measurement after the reception of a stimulus may be influenced by it (cf. Kácha and van der Linden, 2021). In the first experiment, environment related dispositions were operationalized using environmental awareness as a generic variable. In the second experiment, we adjusted the materials based on what we learned from the first study and used the more proximal personal norms as moderator.

## 3 Study 1

### 3.1 Method

Participants were recruited and compensated by a market research institute, aiming for a sample representative of the sociodemographic characteristics of the German population (*N* = 372, *M*_age_ = 45.83, SD_age_ = 14.57, 47% female); 10% have offset a flight in the past. In the first survey (T1), pre-attitudes, behavioral intentions, perceived effectiveness, and environmental awareness were measured. Ten days later (T2), respondents participated in a 3 (descriptive social norm appeal: static vs. dynamic vs. absent) × 2 (injunctive social norm appeal: present vs. absent) between-subjects experiment. Moreover, there was a control group and participants in this group did not receive any stimulus. With the two-wave design, we wanted to ensure that the T1 measurements were no longer salient enough to influence the effect of the stimulus on the dependent variables. Simultaneously, the interval between waves should not be too long, as longer intervals can lead to a higher dropout rate and pre-treatment effect become more likely (Kane, 2024). This research project (including the second study) was approved by the university’s ethics committee (approval no. 21.6.20).

#### 3.1.1 Research subject: voluntary carbon offsetting

To test our hypotheses, we looked for an environmentally friendly behavior that is approved by a majority and performed by an increasing minority of people. We chose voluntary carbon offsetting (VCO) which is a simple bridging mechanism to offset travel emissions by financing climate-friendly projects until full carbon neutrality can be achieved, as full decarbonization is not possible in the short term (United Nations, 2015; Streck, 2021). There seem to be conflicting social norms regarding offsetting behavior and in the European Union, 90% of people believe that greenhouse gas emissions should be reduced to a minimum, while the remaining emissions should be offset (European Commission, 2021). Simultaneously, however, less than 10% engage in VCO themselves (Gössling et al., 2009; Mair, 2011; Choi et al., 2016; Segerstedt and Grote, 2016; Berger et al., 2022). Therefore, VCO seems to be a suitable topic for this study because there seems to be an injunctive majority norm and a descriptive minority norm, both of which can be emphasized by social norm appeals.

#### 3.1.2 Procedure: two-wave study

Only people who had not ruled out flying for personal reasons were included in the study. In the first survey (T1), participants were told that this study concerned carbon offsets and that they would see a video about VCO in the second part. After answering questions about demographics and the frequency of flying, participants were asked about their intentions to offset in the future, their attitude toward VCO, the perceived effectiveness of VCO, and their prior experiences. At the end, participants’ environmental awareness was measured before they were informed that they would be invited to participate in the second part of the study in 10 days.

In the second part (T2), participants were randomly assigned to one of the six experimental conditions or to a control group without stimulus. This was followed by measurements of perceived descriptive and injunctive social norms. Afterwards, their intention to offset a flight, their attitude toward VCO, and perceived effectiveness were again measured as dependent variables. Finally, the participants were debriefed and redirected to the market research institute.

#### 3.1.3 Stimulus material: explainer videos

Online videos are a popular and effective means of communicating climate change issues (Welbourne and Grant, 2015; Ettinger et al., 2021; Seger et al., 2023). Most studies of social norm appeals (especially in relation to sustainable behavior) are based on text stimuli, although social norm appeals embedded in a video message may be more effective in promoting sustainable behavior (Rhodes et al., 2020). Therefore, we used explainer videos to deliver the social norm appeals. Explainer videos are very popular on social media and are often used as sources of information for specific questions and topics (Koch and Bleisch, 2020). These short films explain abstract concepts using narrative techniques and combinations of voiceover and animation (Schorn, 2022). This allows social norm appeals to be communicated across multiple channels, as they can be presented both verbally and visually, which can increase persuasiveness (cf. Rhodes et al., 2020). Explainer videos are effective tools for communicating knowledge, which is useful in the context of VCO (cf. Boy et al., 2020). Many people do not know what carbon offsets are; however, providing them with relevant information increases their willingness to offset (Gössling et al., 2009; Wulfsberg et al., 2016; Lu and Wang, 2018; Denton et al., 2020; Ritchie et al., 2021). Therefore, we used the Simpleshow Video Maker and a professional speaker to produce six whiteboard explainer videos on VCO (2–3 min). All videos explained the compensation and how it worked. At the end of the videos, the different social norm appeals were placed and presented verbally and visually (see Supplementary material).

#### 3.1.4 Manipulation

**Figure 1:**
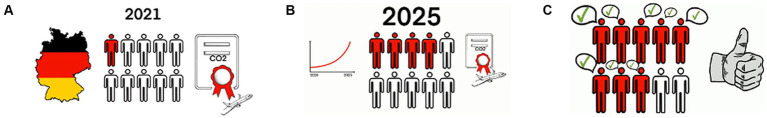
Visualization of descriptive and injunctive social appeals. **(A)** Visualization static descriptive social norm appeal. **(B)** Visualization dynamic descriptive social norm appeal. **(C)** Visualization injunctive majority social norm appeal.

The complete storyboard and the manipulation are included in the Supplementary material. In the static descriptive condition, participants were informed that only one in ten German travelers offset their flights: “When looking at the German population, only one in ten people have voluntarily compensated for their flight in 2021.” In the dynamic condition, participants were informed of an ongoing trend: “When looking at the German population, already one in ten people voluntarily offset their flight in 2020. This number is five times higher than in the previous year. This is a positive trend, and the proportion is expected to quadruple in the coming years.” Experimental conditions including an injunctive appeal stated that the majority of Germans approve of VCO: “Many surveys show that a clear majority of Germans are in favor of offsets: Up to 80% say that voluntary CO_2_ offsets in air travel are sensible, good and important.” In addition to this verbal presentation, social norm appeals were visualized differently (see Figure 1).

#### 3.1.5 Measures

In the first questionnaire, we measured environmental awareness following Geiger and Holzhauer (2020). The intention to offset and attitude toward VCO were measured following Denton et al. (2020). Moreover, we measured the perceived effectiveness of VCO following Choi et al. (2016), because stronger perceived efficacy can relate to a stronger adaptation of the behavior (Van Valkengoed and Steg, 2019). In the second part of the study (T2), these three variables were measured again as persuasive outcomes after participants watched the explainer videos 10 days later. Furthermore, we measured preconformity, perceived descriptive and injunctive social norms. We also measured the perceived quality of the videos (credibility, professionalism, and likeability). All constructs were measured on 5-point scales (see Table 1) and tested in preliminary studies (see Schorn et al., 2023; Schorn and Wirth, 2023).

**Table 1:** Summary of measurement in Study 1.

	T1		T2	
Variable	*M* (SD)	*α*	*M* (SD)	*α*
Intention to offset (5 items)*	2.77 (1.20)	0.97	3.30 (1.24)	0.98
Attitude toward VCO (3 items)*	3.42 (1.01)	0.90	3.84 (1.06)	0.92
Perceived effectiveness of VCO (6 items)*	3.18 (1.09)	0.95	3.52 (1.12)	0.96
Perceived descriptive social norms**	–	–	20.29 (21.72)	–
Perceived trend (preconformity)*	–	–	3.39 (1.19)	–
Perceived injunctive social norms**	–	–	52.10 (25.11)	–
Environmental awareness (14 items)*	3.74 (0.76)	0.93	–	–
Perceived quality of the video (6 items)*	–	–	4.00 (0.89)	0.92

*Measured on 5-point scales. **Measured on scale from 1 to 100%.

## 4 Results

To ensure data quality, we conducted quality checks in the survey (e.g., “Please select the box on the left to show that you have read the question”), and individuals who did not pass were redirected during the survey to prevent questionable research practices (cf. Matthes et al., 2015). In the second part, cases who indicated that they “did not watch the video attentively” or stayed on the stimulus page for more than approximately twice the time of the longest video (350 s) were also excluded to ensure that the stimuli are viewed attentively and can be recalled (Kane, 2024). This excluded 9 cases at T2, leaving 372 valid cases.

### 4.1 Perceived quality

First, we checked the perceived quality and credibility of the videos using analysis of variance (ANOVA) to ensure the external validity of the material. There were no significant differences between the videos, which meant that the videos were all perceived to be equally high quality. In addition, all item means were in the positive part of the semantic differentials and differed significantly from the neutral center of the scale (*M* > 3.00, *p* < 0.001), again demonstrating the high quality of the material.

### 4.2 Control group

To determine whether the videos were effective overall, we compared the experimental groups and the control group without video. Contrast analysis showed that those who had received a video with a social norm appeal reported a higher intention to offset. Regarding the attitudes toward VCO and perceived effectiveness, there were only significant differences for videos including an injunctive appeal (see Supplementary material on OSF).^1^ There were no significant differences between the control group and the video without social norm appeal, suggesting that the social norm appeals had an effect: The video required a social norm appeal to develop the persuasive effect compared to the baseline measurement (see Table 2).

**Table 2:** Contrast analysis of experimental groups vs. control group for the intention to offset.

	*M_T2_*	SD*_T2_*	*t*	*p*
ISNA | static DSNA	3.62	1.06	4.05	<0.001
no ISNA | static DSNA	3.13	1.30	2.61	0.009
ISNA | dynamic DSNA	3.37	1.33	4.21	<0.001
No ISNA | dynamic DSNA	3.50	1.26	2.95	0.003
ISNA | no DSNA	3.47	1.20	3.61	<0.001
No ISNA | no DSNA	3.19	1.22	1.51	0.132
Gender			1.66	0.099
Age			2.50	0.013
T1 intention			18.91	<0.001
Control group (no video)	2.83	1.2		

Analyses are controlled for gender, age, and T1 intentions. DSNA, descriptive social norm appeal; ISNA, injunctive social norm appeal.

### 4.3 Hypotheses testing

To test the hypotheses, we conducted analyses of covariance (ANCOVAs) and multivariate analyses of covariance (MANCOVAs), controlling for gender, age, and T1 measurement (for persuasive outcomes). Covariates were selected on theoretical grounds because these variables generally predict sustainable behavior (e.g., Bamberg et al., 2007; Meyer, 2015; Vicente-Molina et al., 2018; Wang et al., 2021).^2^

#### 4.3.1 Perceived descriptive social norms

**Figure 2:**
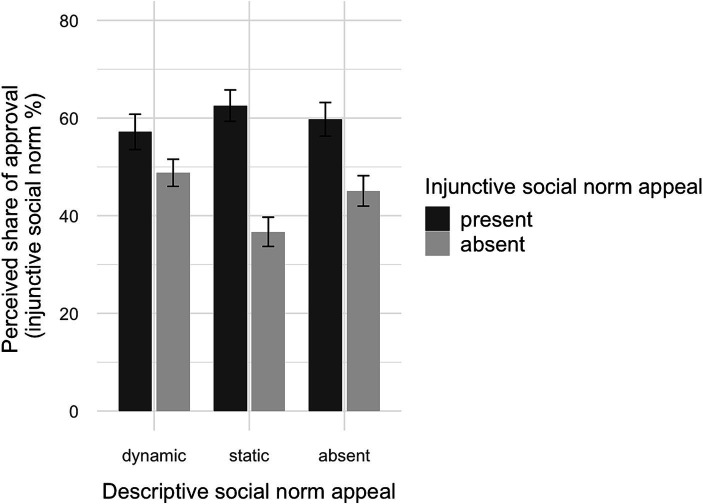
Interaction plot of descriptive and injunctive social norm appeals on perceived descriptive social norms.

Regarding the perceived prevalence of behavior, ANCOVA showed a main effect of the descriptive (*F*(2, 309) = 24.91, *p* < 0.001, *η*^2^ = 0.14) and the injunctive conditions (*F*(1, 309) = 51.59, *p* < 0.001, *η*^2^ = 0.14), and an interaction effect (*F*(2, 309) = 10.21, *p* < 0.001, *η*^2^ = 0.06). Participants reported a higher share of people offsetting their flights in the condition without descriptive appeal (*M*_adj_ = 30.07, CI [26.58, 33.55]) than in the dynamic (*M*_adj_ = 17.43, CI [13.81, 21.04], *p* < 0.001) or static condition (*M*_adj_ = 12.9, CI [9.38, 16.43], *p* < 0.001). Moreover, participants receiving an injunctive appeal (*M*_adj_ = 27.78, CI [24.9, 30.66]) reported a higher share than participants not receiving an injunctive appeal (*M*_adj_ = 12.69, CI [9.81, 15.58]), indicating a spillover effect (see Figure 2). As the interaction between descriptive and injunctive appeals is ordinal, the data support H1a, and a message highlighting the minority of people engaging in VCO (static and dynamic appeals) negatively affects the perceived prevalence of the behavior.

Regarding the perception of a trend, there was an effect between the descriptive conditions (*F*(2, 311) = 9.94, *p* < 0.001, *η*^2^ = 0.06), but not between the injunctive conditions (*p* > 0.71) or their interaction (*p* > 0.14). Participants who received a dynamic appeal perceived a stronger trend (*M*_adj_ = 3.81, CI [3.58, 4.04]) than those who received a static appeal (*M*_adj_ = 3.21, CI [2.99, 3.43], *p* < 0.001) or no descriptive appeal (*M*_adj_ = 3.16, CI [2.94, 3.38], *p* < 0.001). Thus, H2a and H3a are supported because a dynamic appeal leads to a stronger perception of a trend than a static minority appeal or a message without a descriptive appeal.

#### 4.3.2 Perceived injunctive social norms

**Figure 3:**
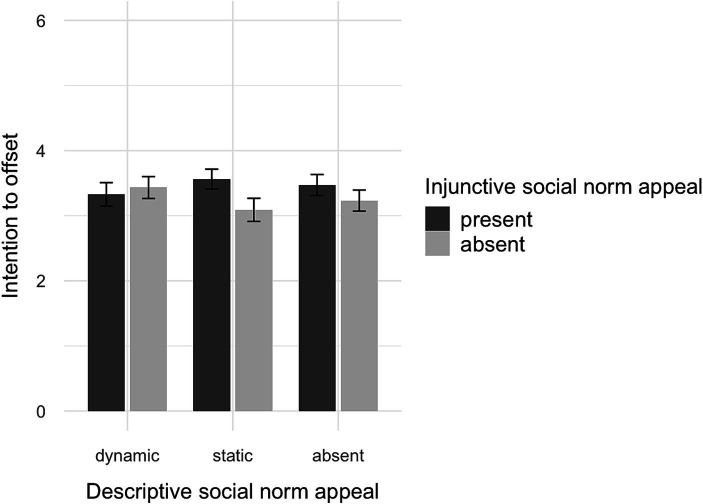
Interaction plot of descriptive and injunctive social norm appeals on perceived injunctive social norms.

As expected, the ANCOVA showed an effect for the injunctive conditions (*F*(1, 309) = 42.43, *p* < 0.001, *η*^2^ = 0.12), but not for the descriptive conditions (*p* = 0.45). Participants receiving an injunctive appeal indicated a higher share of people approving VCO (*M*_adj_ = 60.76, CI [57.1, 64.43]) than those without an injunctive appeal (*M*_adj_ = 43.48, CI [39.81, 47.16]). Therefore, H4a is supported because an injunctive appeal increased the perceived prevalence of approval. Moreover, there was a weak but significant interaction between descriptive and injunctive appeals (*F*(2, 309) = 3.15, *p* = 0.04, *η*^2^ = 0.02, see Figure 3). Differences between the injunctive conditions were only significant when combined with a static appeal (*p* < 0.001) or no descriptive appeal (*p* < 0.01) but not when combined with a dynamic appeal (*p* = 0.31).

#### 4.3.3 Persuasive outcomes

We conducted a MANCOVA for the intention to offset, attitude toward VCO, and perceived effectiveness, controlling for gender, age, and T1 measurements. There was a significant effect of the injunctive appeal (Wilk’s Λ (3, 306) = 0.97, *p* = 0.02, *η*^2^ = 0.03) but not for the descriptive conditions (*p* = 0.11) or the interaction (*p* > 0.54).

**Figure 4:**
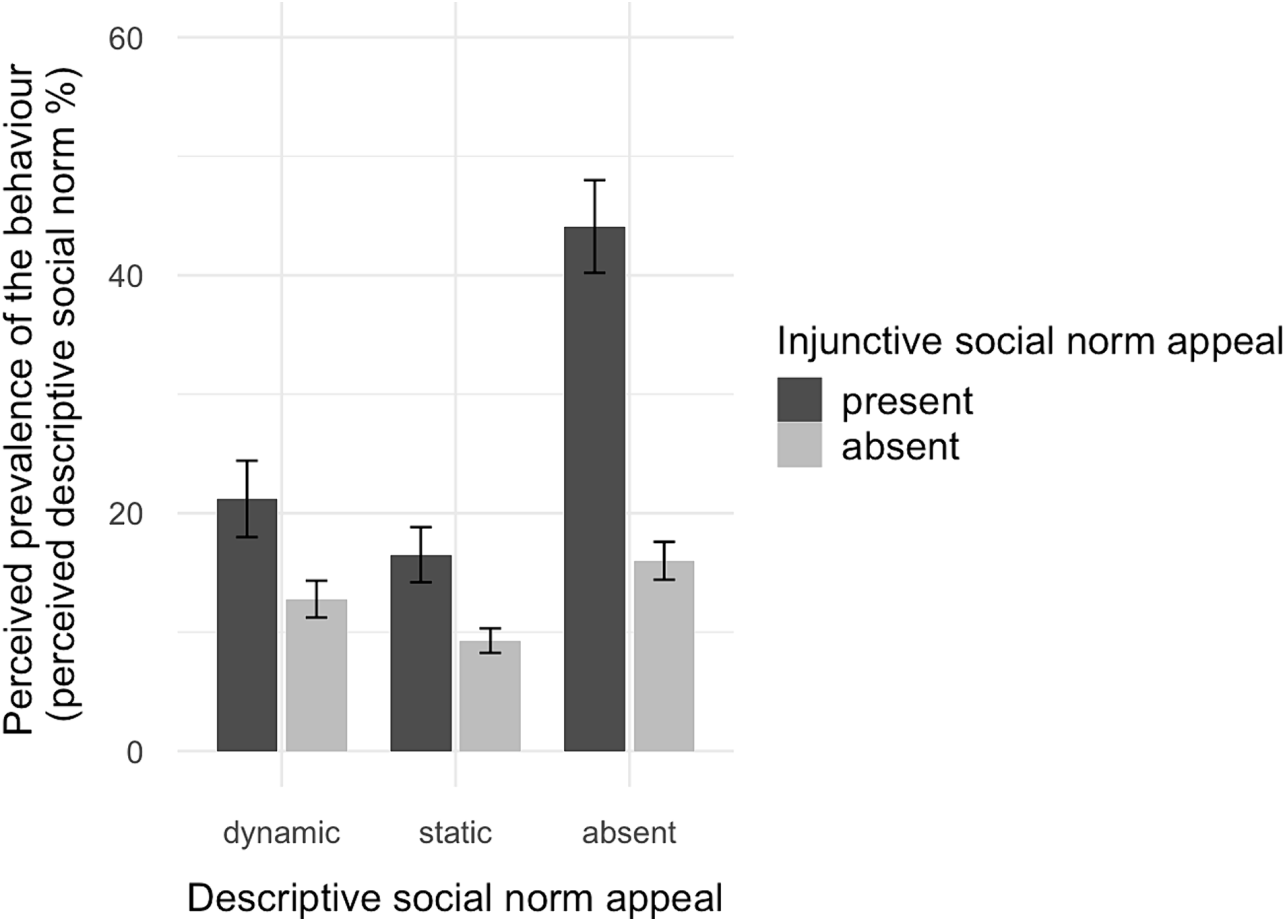
Interaction plot of descriptive and injunctive social norm appeals on the intention to offset.

Separate analyses showed that there was a significant effect of the injunctive appeal on the intention to offset (*F*(1, 310) = 7.42, *p* < 0.01, *η*^2^ = 0.02). Participants who received an injunctive appeal reported a higher intention (*M*_adj_ = 3.51, CI [3.38, 3.65]) than those who did not receive an injunctive appeal (*M*_adj_ = 3.24, CI [3.11, 3.38]). The effects of the descriptive conditions (*p* > 0.31) or the interaction (*p* > 0.86) were not significant (see Figure 4). Regarding attitude toward VCO, there was a significant effect of the injunctive appeal (*F*(1, 310) = 8.46, *p* = 0.004, *η*^2^ = 0.03). Participants who received an injunctive appeal reported a higher attitude (*M*_adj_ = 4.02, CI [3.89, 4.14]) than those who did not receive an injunctive appeal (*M*_adj_ = 3.76, CI [3.63, 3.88]). The effects of the descriptive conditions (*p* > 0.76) or their interaction (*p* > 0.41) were not significant. Moreover, we found an effect of the injunctive appeal on perceived effectiveness (*F*(1, 310) = 5.16, *p* = 0.02, *η*^2^ = 0.02), and receiving an injunctive appeal improved perceived effectiveness (*M*_adj_ = 3.66, CI [3.53, 3.8]) compared with not receiving an injunctive appeal (*M*_adj_ = 3.45, CI [3.31, 3.58]). There was no effect of the descriptive conditions (*p* = 0.11) or the interaction (*p* > 0.95).

Overall, considering the persuasive effects, H4b is supported owing to the positive effect of the injunctive appeal. However, the variations in the descriptive condition had no effect, resulting in the rejection of H1b, H2b, and H3b. Regarding RQ2, we found no interaction effects: There was no evidence of social norm conflict and no improvement by combining an injunctive majority appeal and a dynamic descriptive appeal.

#### 4.3.4 Environment related dispositions

To test the differences depending on recipients’ pro-environmental dispositions, we included environmental awareness as a moderator (median split = 3.86). MANOVA of persuasive outcomes found a significant effect of the injunctive appeal (Wilk’s Λ (3, 300) = 0.97, *p* = 0.03, *η*^2^ = 0.03) but no effect between the descriptive conditions (*p* > 0.11). Moreover, there was no interaction between environmental awareness and the descriptive conditions (*p* > 0.95) or the injunctive conditions (*p* > 0.38). The results did not differ when including environmental awareness as a metric moderator or other splits, for example, based on the standard deviation, or considering the dependent variables in separate ANOVAs. Therefore, H5 must be rejected because people with weak pro-environmental dispositions are not more prone to the impact of social norm appeals.

### 4.4 Discussion

The results of the first study show that explainer videos, including social norm appeals, can influence perceived social norms but have limited influence on persuasive outcomes. A injunctive majority appeal increased persuasive outcomes, but there were no differences between descriptive conditions and no differences depending on individuals’ environmental awareness. Regarding the research question about the influence of conflicting social norm appeals on perceived norms, we found that the spillover effects of the injunctive majority appeal and perceived descriptive norms were higher overall when an injunctive appeal was included. However, there were no spillover effects on perceptions of a trend or perceived injunctive norms. Nevertheless, the static descriptive minority appeal combined with the injunctive majority appeal had a positive influence on perceived injunctive social norms, which is surprising. Minority descriptive appeals may emphasize the urgency of action, which appears to increase perceived approval percentages (cf. Habib et al., 2021).

## 5 Study 2

We drew lessons from the results of the first study to improve materials for the second study. As the social norm appeals were embedded in longer videos, the manipulation was only one small part of a complex media stimulus, including a large amount of information apart from the social norm appeals. In other studies, social norm appeals are often much more present, if not standalone (e.g., on a poster, especially in field studies, e.g., Loschelder et al., 2019). Therefore, we included verbal repetitions of the different social norm appeals earlier in the video and title to increase salience. Other studies applying dynamic descriptive appeals do not mention the baseline frequency in this condition but only state that “more and more” people are joining the target behavior (e.g., Loschelder et al., 2019). It is possible that 10% participation in VCO was too small to evoke a preconformity even if a trend is mentioned because this is still far from the threshold of 50%. Therefore, we revised the manipulation of the dynamic descriptive appeal and removed the baseline of 10% participation in the second study. Regarding the effect of environment related dispositions, environmental awareness may be too distal a construct, so we replaced this construct in the following study with personal norms regarding VCO.

### 5.1 Method

The second experiment followed the procedure described above. However, some adjustments were made (see Supplementary material). First, we added written titles at the beginning of each video that address the manipulation of the different social norm appeals to increase the salience of the social norm appeals within the explainer videos (e.g., “Why only a few people compensate for their air travel”). Verbal teasers with different appeals were also included earlier in the video. Second, the manipulation of the dynamic descriptive appeal was adjusted, and the baseline of 10% participation in VCO was removed (“But how much is offset in Germany? A strong trend is emerging: More and more people are choosing to offset their air travel”). The changes in the text meant that the audio tracks of the videos had to be rerecorded, resulting in a change in speaker. Moreover, the distal construct of environmental awareness was replaced with personal norms as a more proximal construct, and feelings of obligation to deal with the environmental impact of air travel were measured in the first wave. Furthermore, we included perceived social norms and subjective norms in the first questionnaire, which allowed us to draw more detailed conclusions about the changes in perceived social norms evoked by the stimuli (see Table 3). All variables were measured on 7-point scales to increase accuracy and variance (Dawes, 2008; Finstad, 2010). As the results of the first study showed that the effects were small (particularly with regard to possible interactions), we increased the sample size to allow more nuanced analyses with mediators and moderators. Finally, we asked participants whether they would be willing to calculate the price of compensation for a past flight to measure their actual behavior. We implemented a tool in the questionnaire, and if participants agreed to calculate the price for VCO, they were asked to enter the departure and arrival airports, which were used to calculate the price based on distance. Afterward, they were shown the price and asked how likely it was that they would compensate for that flight (willingness to pay for VCO: “For your flight from [start] to [destination] ([distance] km, economy class), offsetting the emissions would cost about [price] Euro. How likely are you to offset your air travel at this price?”).

**Table 3:** Summary of measurement in Study 2.

	T1		T2	
Variable	*M* (SD)	*α*	*M* (SD)	*α*
Intention to offset (5 items)*	3.74 (1.95)	0.98	3.95 (2.10)	0.99
Attitude toward VCO (3 items)*	4.76 (1.69)	0.93	4.93 (1.79)	0.93
Perceived effectiveness of VCO (6 items)*	4.05 (1.63)	0.95	4.20 (1.78)	0.96
Calculation of price for past flight (yes)	–	–	59.52%	–
Willingness to pay for VCO (*n* = 400)*	–	–	5.17 (1.86)	–
Personal norms on VCO*	4.13 (1.77)	0.90	–	–
Perceived descriptive social norms***	25.55 (21.04)	–	23.41 (20.28)	–
Perceived trend (preconformity)*	4.53 (0.99)	–	4.53 (0.94)	–
Perceived injunctive social norms***	37.93 (23.89)	–	38.26 (23.82)	–
Subjective norms***	16.05 (23.01)	–	14.64 (21.89)	–
Perceived quality of the video (6 items)**	–	–	4.04 (0.91)	0.92

*7-point scale. **5-point scale. ***Measured on scale from 1 to 100%. The willingness-to-pay was only asked of participants who had previously agreed to calculate the price of offsetting a past flight.

Participants were recruited from the same market research institute (*M*_age_ = 49.41, SD_age_ = 14.52, 52% female). Individuals who participated in Study 1 were unable to participate in Study 2. Participants who did not pass the quality checks were excluded in the same manner as those in the first study. This excluded 288 cases; after merging the data from the first and second questionnaires, 1,378 valid cases remained.

### 5.2 Results

Before testing the hypotheses, we compared perceived social norms with actual behaviors and attitudes. On average, people estimated that 38.26% of the population approved of VCO. Simultaneously, however, 66.30% at least agreed somewhat with the statement to approve of VCO, and 29.76% fully agreed that they approved of VCO. Regarding descriptive norms, the observation was the opposite; the proportion of people who currently compensated for flights was estimated to be 23.41%. Simultaneously, however, only 14.40% stated that they have ever compensated for a flight themselves. Therefore, the injunctive majority norm was underestimated, whereas the descriptive minority norm was overestimated. At the same time, individuals systematically underestimated the attitude–behavior gap for other people (38% vs. 23%) as compared to the actual attitude–behavior gap (66% vs. 14%). This is an interesting finding; it seems that people do not believe that others behave as inconsistently as they do themselves. In line with results from Andre et al. (2024) there seems to be a prevailing pessimism regarding others’ support for climate action which could deter individuals from engaging in climate action. Moreover, these results show that in the case of VCO there are indeed conflicting social norms: The majority support VCO (injunctive majority), but only a minority engages in VCO themselves (descriptive minority).

#### 5.2.1 Perceived quality

There were no significant differences between videos regarding perceived quality or credibility (*M* = 4.04, SD = 0.91). All item means were significant in the positive part of the semantic differential for all videos (*M* > 3.00, *p* < 0.001).

#### 5.2.2 Control group

Contrast analysis showed that people who received a video, regardless of the social norm appeals, reported an increased intention to offset and perceived effectiveness (see Supplementary material on OSF). Regarding attitudes, there were only (at least marginal) differences between the videos, including any social norm appeal.

#### 5.2.3 Hypotheses testing

To test the hypotheses, we conducted (M)ANCOVAs, controlling for age, gender, and T1 measurements.

##### 5.2.3.1 Perceived descriptive social norms

Regarding the perceived prevalence of the behavior, ANCOVA showed a main effect for the descriptive (*F*(2, 1,005) = 68.85, *p* < 0.001, *η*^2^ = 0.12) and the injunctive conditions (*F*(1, 1,005) = 93.43, *p* < 0.001, *η*^2^ = 0.09), and an interaction effect (*F*(2, 1,005) = 25.75, *p* < 0.001, *η*^2^ = 0.05). Participants reported a higher share in the condition without descriptive appeal (*M*_adj_ = 35.23, CI [32.93, 37.54]; *p* < 0.001) and the dynamic condition (*M*_adj_ = 32.28, CI [29.9, 34.66]; *p* < 0.001) than in the static condition (*M*_adj_ = 17.16, CI [14.84, 19.48]). Moreover, participants receiving an injunctive appeal (*M*_adj_ = 35.00, CI [33.08, 36.93]) reported a higher share than participants not receiving an injunctive appeal (*M*_adj_ = 21.66, CI [19.78, 23.55]), again showing a spillover effect. As the interaction between the descriptive and injunctive conditions is ordinal, the data again support H1a, and a message highlighting the minority of people engaging in VCO (static descriptive appeal) negatively affects the perceived prevalence of the behavior.

Regarding the perception of a trend, there was an effect of the descriptive conditions (*F*(2, 1,005) = 38.41, *p* < 0.001, *η*^2^ = 0.07), but not of the injunctive appeal (*p* >. 24) or their interaction (*p* > 0.09). Participants who received a dynamic descriptive appeal perceived a stronger trend (*M*_adj_ = 5.32, CI [5.21, 5.43]) than those who received a static appeal (*M*_adj_ = 4.75, CI [4.64, 4.85], *p* < 0.001) or no descriptive appeal (*M*_ad_ = 4.72, CI [4.61, 4.82]). Thus, H2a and H3a are supported because a dynamic descriptive appeal led to a stronger perception of a trend than a static descriptive minority appeal or a message without a descriptive appeal.

##### 5.2.3.2 Perceived injunctive social norms

An ANCOVA showed an effect for the injunctive appeal (*F*(1, 1,005) = 261.71, *p* < 0.001, *η*^2^ = 0.21), the descriptive conditions (*F*(2, 1,005) = 4.52, *p* = 0.01, *η*^2^ < 0.01), and their interaction (*F*(2, 1,005) = 6.66, *p* = 0.001, *η*^2^ = 0.01). Participants receiving an injunctive appeal indicated a higher share of people approving VCO (*M*_adj_ = 64.79, CI [62.8, 66.78]) than those not receiving an injunctive appeal (*M*_adj_ = 41.74, CI [39.8, 43.69]). Therefore, H4a is supported, and an injunctive majority appeal increases the perceived prevalence of approval.

Moreover, there was a small spillover effect, and a static descriptive appeal (*M*_adj_ = 50.09, CI [47.7, 52.49]) resulted in a lower estimate of approval than messages without a descriptive appeal (*M*_adj_ = 55.24, CI [52.86, 57.61]; *p* = 0.02). The differences to the dynamic appeal were not significant (*M*_adj_ = 53.72, CI [51.27, 56.18], *p* > 0.16). Regarding the interaction, post-hoc analysis revealed no differences between the descriptive conditions when an injunctive appeal was present (*p* > 0.58). However, when there was no injunctive appeal, the static descriptive appeal performed worse than the dynamic descriptive appeal (*p* < 0.01) and the condition without a descriptive appeal (*p* < 0.001). Thus, when an injunctive appeal was present, perceived injunctive norms were not affected by the descriptive appeal; however, when there was no injunctive appeal, the baseline in the static appeal seemed to cause an anchor effect. Moreover, the share of approval was estimated to be lowest in the static descriptive condition without an injunctive appeal (*M*_adj_ = 34.86, CI [31.37, 38.35]) and highest when an injunctive appeal was included in addition to the static descriptive appeal (*M*_adj_ = 65.64, CI [61.98, 69.30]; *p* < 0.001).

##### 5.2.3.3 Persuasive outcomes

A MANCOVA was performed for intention to offset, attitude toward VCO, and perceived effectiveness, controlling for age, gender, and T1 measurements. There were no significant effects of the injunctive conditions (*p* = 0.77), the descriptive conditions (*p* = 0.63) or their interaction (*p* = 0.88). When considering actual behavior (calculating the price to offset a past flight), logistic analyses did not reveal any significant effects of the descriptive conditions (*p* = 0.20), the injunctive conditions (*p* = 0.54) or their interaction (*p* = 0.90). In the next step, we only looked at cases that agreed to calculate the price for VCO and conducted another ANOVA using the willingness to pay as the dependent variable, controlling for gender, age, and price. Again, there were no significant effects of the descriptive conditions (*p* = 0.28), the injunctive conditions (*p* = 0.32), or their interaction (*p* = 0.93). Therefore, we reject Hypotheses H1b, H2b, H3b, and H4b.

##### 5.2.3.4 Environment related dispositions

To test for differences depending on the recipients’ environment related dispositions, we included personal norms as a moderator (median split = 4.50). The MANOVA of the persuasive outcomes found no effects between the descriptive conditions (*p* = 0.54), the injunctive conditions (*p* = 0.79), or their interactions (*p* = 0.88). Moreover, there were no interactions between personal norms and the descriptive conditions (*p* = 0.87) or the injunctive conditions (*p* = 0.09). The results are the same when including personal norms as a metric moderator or other splits, for example, based on the standard deviation, or considering the dependent variables separately in ANOVAs. Therefore, H5 is rejected again because there are no significant effects when personal norms are used as a moderator.

#### 5.2.4 Moderated mediation analyses

We did not observe any direct effects of descriptive or injunctive social norm appeals on behavioral outcomes in this study. However, several other studies concluded that these effects may be mediated by perceived social norms (e.g., Chung and Lapinski, 2019; Loschelder et al., 2019; Schorn and Wirth, 2023). Therefore, we tested the mediation of perceived social norms using PROCESS model 8 (Hayes, 2018). We used 1,000 bootstrapped samples to estimate 95% confidence intervals of the indirect paths. Age, gender and the T1 measurement were again used as covariates.

First, perceived future descriptive norm (preconformity) was included as a mediator, and the intention to offset as dependent variable. Descriptive norm appeal (1 = dynamic, 0 = static) was entered as independent variable and the injunctive appeal (0 = absent, 1 = present) as moderator. The results show that the dynamic appeal indirectly influenced the intention to offset through its effect on preconformity. Participants who received a dynamic appeal anticipated a greater trend than those who received the static minority appeal (*B* = 0.42, *p* < 0.001). Effects of the injunctive appeal were not significant (*p* = 0.34). Furthermore, there was a significant interaction effect between descriptive and injunctive appeals (*B* = 0.36, *p* = 0.02): When participants received an injunctive appeal, there was a stronger effect between the descriptive conditions (*B* = 0.78, *p* < 0.001) than when receiving no injunctive appeal (*B* = 0.42, *p* < 0.001). There were no direct effects of the descriptive (*p* = 0.09), the injunctive conditions (*p* = 0.76) or their interaction (*p* = 0.67) on the intention to offset. However, participants who perceived a stronger trend reported a stronger behavioral intention (*B* = 0.40, *p* < 0.001). This indirect effect of the dynamic descriptive appeal was stronger when there was an injunctive appeal (*B* = 0.31, 95% CI [0.20–0.46]) than without an injunctive appeal (*B* = 0.17, 95% CI [0.08, 0.29]).

Next, we used perceived injunctive social norms as mediator and entered the injunctive norm appeal as independent variable and the descriptive appeal as moderator. Again, the results show that the social norm appeals indirectly influenced the intention to offset through its effect on perceived injunctive social norms. Participants who received an injunctive appeal anticipated a greater prevalence of approval than those not receiving an injunctive appeal (*B* = 29.71, *p* < 0.001). Moreover, there was an effect of the descriptive appeal and participants receiving a dynamic appeal reported a stronger perceived injunctive social norm than participants receiving a static appeal (*B* = 9.67, *p* < 0.001). Furthermore, there was a significant interaction effect between descriptive and injunctive appeals (*B* = −12.43, *p* < 0.001): When participants received a static appeal, there was a stronger effect of the injunctive appeal (*B* = 29.71, *p* < 0.001) than when receiving a dynamic appeal (*B* = 17.28, *p* < 0.001). There were no direct effects of the injunctive conditions (*p* = 0.06), the descriptive conditions (*p* = 0.18) or their interaction (*p* = 0.08) on the intention to offset. However, participants who perceived greater future injunctive norms reported a stronger behavioral intention (*B* = 0.01, *p* < 0.001). This indirect effect of the injunctive appeal was stronger when there was a static descriptive appeal (*B* = 0.32, 95% CI [0.17–0.50]) than a dynamic descriptive appeal (*B* = 0.19, 95% CI [0.09, 0.30]).

### 5.3 Discussion

In the second study, we found the hypothesized effects of descriptive and injunctive appeals on perceived social norms, but there were no effects of descriptive and injunctive social norm appeals, or their interaction on persuasive outcomes. Therefore, we were unable to replicate the effects of the injunctive appeal from the first study. Furthermore, we did not find a moderating effect of personal norms. Therefore, we cannot confirm the results of Schultz et al. (2016), de Groot et al. (2021), and Carfora et al. (2022), who demonstrate that social norm appeals work differently depending on individuals’ personal norms regarding the topic. Nevertheless, the effects on behavioral intention could be mediated through perceived social norms: The results of the moderated mediation analyses show that social norm appeals can increase perceived social norms, and this strengthens behavioral intentions. Consequently, modifying the perception of social norms can lead to a change in subsequent beliefs and behavioral intentions.

## 6 General discussion

This research examined the effects of conflicting social norm appeals on perceived social norms, persuasive outcomes, and the role of individuals’ environment related dispositions. Overall, we found strong effects of social norm appeals on perceived social norms, but not on persuasive outcomes. Furthermore, there were no interactions between environmental awareness or personal norms, and the social norm appeals (see Table 4). Nevertheless, these results have several relevant implications for research on social norm appeals.

**Table 4:** Overview of hypothesis testing.

		Study 1	Study 2
H1	A message including a static descriptive minority appeal (a) negatively affects the perceived prevalence of the behavior and (b) is less persuasive than a message without a descriptive minority appeal.	(a) supported (b) rejected	(a) supported (b) rejected
H2	A message including a dynamic descriptive norm appeal (a) leads to a stronger perception of a trend and (b) is more persuasive than a message including a static descriptive minority appeal.	(a) supported (b) rejected	(a) supported (b) rejected
H3	A message including a dynamic descriptive norm appeal (a) leads to a stronger perception of a trend and (b) is more persuasive than a message without a descriptive appeal.	(a) supported (b) rejected	(a) supported (b) rejected
H4	A message including an injunctive majority appeal (a) increases the perceived prevalence of approval and (b) is more persuasive than a message without an injunctive majority appeal.	(a) supported (b) supported	(a) supported (b) rejected
H5	The effects of different social norm appeals are more pronounced in people with weaker environment related dispositions than in those with strong ones.	rejected	rejected

The first important finding is that the social norm appeals exerted strong effects on perceived social norms, suggesting that the experimental manipulation worked and social norm appeals could influence perceived norms, at least in the short term. In addition to the hypothesized effects of the social norm appeals on perceived norms, we found spillover effects, and the injunctive majority appeal also increased perceived descriptive norms, which is consistent with other research (Eriksson et al., 2015; Rhodes et al., 2020). The anchoring effect of the injunctive majority appeal was similar in strength to that of the descriptive appeal. However, the static descriptive minority appeal decreased perceived injunctive norms compared with the condition without a descriptive appeal in the second study, but the effect was relatively weak and there were no differences between static and dynamic descriptive appeals. Therefore, we found mostly positive spillover effects of injunctive majority appeal, but few negative spillover effects of the static descriptive minority appeal.

These effects on perceived social norms are important because individuals systematically misperceive social norms (e.g., Boon-Falleur et al., 2022; Andre et al., 2024). Our results show that communicating the injunctive majority of people approving of climate action could be a powerful intervention to correct these misperceptions. Before participants saw the social norm appeals (T1), perceived injunctive norms were underestimated, while descriptive social norms were overestimated. The injunctive majority appeal could correct this misestimation of injunctive norms. Moreover, the use of injunctive majority appeals could also help to increase perceived descriptive norms. Descriptive norm appeals could correct perceived descriptive norms as well (when they stand alone); however, this appears to be a disadvantage in the case of minority behavior. Thus, these results suggest that injunctive majority appeals in particular can be useful in changing perceived norms in the desired sense. This is an important finding because countries with a stronger approval of pro-climate social norms pass more climate-change-related laws and policies, and the provision of common goods also crucially depends social norms (cf. Andre et al., 2024).

When examining persuasive effects, we found a positive effect of the injunctive appeal in the first study, and the injunctive majority appeal increased the intention to offset, attitude toward VCO, and perceived effectiveness of VCO, independent of the descriptive appeals. However, this effect was not replicated in the second study. This was unexpected and requires explanation as we did not change the text or visualization of the injunctive appeal. Furthermore, we used a very similar operationalization in another study of VCO, wherein we also found a positive effect of the injunctive appeal (Schorn and Wirth, 2023). When participants’ characteristics were examined, no systematic pattern was found between environmental awareness across studies that could explain the results. Moreover, the videos from the different studies did not differ in perceived quality, but changing the speaker might have had an effect. However, some time passed between data collection. Study 1 was conducted approximately 1 year before Study 2 and, at that time, there were still travel restrictions due to the COVID-19 pandemic. When Study 2 was conducted, the focus of media coverage had changed again and environmental issues and climate activism had become more prominent. It is possible that a strong media focus on environmental issues has reduced the scope for changes in attitudes and behavioral intentions.

Regarding the descriptive appeals, we found no differences between the conditions in both studies, suggesting that there were no negative backfire effects of static descriptive minority appeals but also no positive effects of dynamic descriptive appeals. The results did not differ depending on whether the 10% baseline was mentioned or not. However, other studies have been conducted on minority behaviors that are more prevalent than VCO (e.g., Sparkman and Walton, 2017; Mortensen et al., 2019). On the one hand, 10% participation in the VCO could be too little to conform to; on the other hand, this share might seem legitimate, even if the behavior is approved by the majority, and therefore does not cause a social norm conflict (Schorn and Wirth, 2023).

Overall, our research is not alone in these findings on descriptive norm manipulations, and the results of other recent studies on descriptive social norm appeals are more modest regarding the positive effects of dynamic descriptive appeal compared with previous studies (e.g., Sparkman et al., 2020; Boenke et al., 2022; Chung and Lapinski, 2023; Schorn and Wirth, 2023). In particular, when comparing dynamic appeals to a control group without descriptive appeal (compared with static minority appeals), dynamic appeals appear to be less effective (DellaValle and Zubaryeva, 2019; He et al., 2019; Carfora et al., 2022). Moreover, there are studies in the context of normative feedback that suggest that effects of normative feedback could be generally overestimated (Verkooijen et al., 2015; Alblas et al., 2023). Therefore, backfire effects of social norm manipulation in the context of minority behavior do not appear to be as critical as initially assumed.

Regarding the alignment of social norm appeals, it is an important finding that we found no evidence of conflict between social norms in either study. The combination of an injunctive majority appeal and a static descriptive minority appeal did not result in undesirable (backfire) effects, which is consistent with other recent studies that did not show the negative effects of combining minority and majority social norm appeals (e.g., Habib et al., 2021; Schorn and Wirth, 2023). However, we did not examine social norm conflict in a full design (no injunctive minority appeal, no descriptive majority appeal) and therefore can only draw limited conclusions. Backfire effects could be particularly likely if participants are told that most people act sustainably but only a minority approves of that behavior. However, this does not apply to VCO and we did not test this for ethical reasons and because it could have raised credibility concerns. Additionally, the order of conflicting social norm appeals may be important. Habib et al. (2021) suggested that presenting the injunctive majority before the descriptive minority could increase the importance of action and therefore have rather positive effects. However, because we found only a few positive effects of social norm appeals, the lack of social norm conflict should not be viewed as a major achievement.

Several other reasons could explain why we did not find any differences in persuasive outcomes between the experimental conditions. First, the effects might have been weakened because we did not measure environmental behavior. We used the willingness to calculate the price for offsetting the emissions of a past flight as a proxy, but only a little time had to be spent on it, and no financial costs were incurred. Melnyk et al. (2019) suggested that descriptive and injunctive appeals operate differently, depending on whether actual behavior, behavioral intentions, or attitudes are the dependent variables. Descriptive norm appeals may have stronger effects on actual environmental behavior, whereas behavioral intentions and attitudes may be more affected by the injunctive appeals (Melnyk et al., 2019). Therefore, it is possible that the descriptive appeals in our study were unable to exert their full effects because no environmental behaviors were measured.

Furthermore, the target behavior as a research subject may have influenced the results. Lee and Liu (2023) found no significant difference between those who saw a static descriptive appeal and those who were not exposed to the message (control) in their intention to get the flu shot. However, with regard to reducing red meat consumption, those who saw a static descriptive appeal had significantly lower intentions to reduce red meat consumption than those who were not exposed to any message. Therefore, backfire effects occurred with meat consumption but not with vaccinations. One reason for this could be that flu vaccination is a private and invisible behavior, while meat consumption is a social and visible behavior, very connected to an individuals identity. In general, conflicting social norm appeals may have stronger impacts in public contexts because such contexts increase sensitivity to norms (Habib et al., 2021). Offsetting a flight is usually not socially visible; thus, there may not be enough motivation to conform to a descriptive appeal, whether dynamic or static (cf. Moscovici and Personnaz, 1980). In addition, VCO would cost money; therefore, the decision is more conscious than spontaneous, which is why social proof plays a smaller role as a heuristic than in studies in which, for example, a coffee cup is selected at the same price.

Moreover, the social norm appeals were embedded in the explainer videos and the participants were instructed to watch this video carefully, which may have led to in-depth processing. Especially when higher levels of cognitive elaboration are triggered, descriptive appeals can be less effective than injunctive appeals, which can affect the impact of the social norm appeals embedded in an explainer video because it is precisely the aim of explainer videos to impart knowledge that should lead to elaborate processing (Melnyk et al., 2019; Schorn and Wirth, 2023). This could have further weakened effects of the descriptive appeals, assuming that descriptive appeals rather work heuristically or nonconsciously, while injunctive appeals need more elaboration to consciously make the “right” choice (cf. Göckeritz et al., 2009; Melnyk et al., 2019). Nevertheless, the persuasive effects of the injunctive appeal found in the first study were also rather small.

Furthermore, videos explaining why VCO is an important tool for mitigating climate change would have activated personal norms in all experimental groups regardless of their respective appeal. Kácha and van der Linden (2021) suggested that simply activating participants’ moral norms can eliminate the effect of the descriptive norm appeals on environmental behavior. Their operationalization of moral norms lies somewhere between our environmental awareness and personal norms, because they measured the moral obligation to act in an environmentally friendly manner in general. Similar to our results, they found no interaction effects between personal norms and minority versus descriptive majority appeals. However, they found differences between minority and descriptive majority appeals when personal norms were measured after the presentation of the stimulus, but no differences when personal norms were measured before the presentation of the stimulus. Therefore, activating participants’ moral norms could eliminate or suppress the effects of the descriptive appeals. Furthermore, when examining red meat consumption and flu vaccinations, Lee and Liu (2023) found no differences between static descriptive minority appeals and dynamic descriptive appeals, which could also be due to activation of personal norms. They supplemented their descriptive appeal with a direct appeal (e.g., “Get your flu shot”), which was done in an earlier study by Loschelder et al. (2019), which also led to considerably smaller differences between dynamic and static descriptive appeals. All our videos ended with the statement that VCO is one important solution to protect our planet and climate and that together we can ensure that our children can continue to marvel at spectacular glaciers. It is possible that the impact of the descriptive appeals was overridden or weakened because personal norms were activated by the video in general and by the conclusion. This may explain why we did not find any backfire effects of the static descriptive minority appeal or positive effects of the dynamic descriptive appeal. However, we did not measure activation of personal norms after participants were exposed to the stimulus.

The fact that only perceived social norms are influenced and not behavioral intentions may sound sobering at first, but it is possible that a change in perceived norms leads to a change in behavior in the long term. Studies showing the positive effects of dynamic descriptive appeals suggest that dynamic descriptive appeals may require more repetitions to be internalized and have their effects, and that a single exposure to the stimulus may not be sufficient to change behavioral intentions (e.g., Kormos et al., 2015; Berger et al., 2022). Other research suggests that personal norms, which are often a strong predictor of behavior, are internalized social norms (cf. Thøgersen, 2006; Cialdini and Jacobson, 2021). According to this reasoning, repeated exposure to social norm appeals could lead to a stable change in perceived social norms, which could alter personal norms and produce longer-term behavioral changes (cf. Onwezen et al., 2013; Kim and Seock, 2019).

Overall, the effects of social norm appeals on perceived norms and persuasive outcomes could be enhanced when social norm appeals embedded in explainer videos are viewed in real-life settings such as on social media. Viewing an explainer video on social media platforms is only one part of a fragmented media environment and is not independent of other content from which social norm appeals could benefit. For example, Spartz et al. (2017) demonstrated that even the number of likes of a YouTube video can change the perceived importance (salience) of climate change among “most Americans.” Young and Jordan (2013) concluded that pictures on social media can influence health behaviors because they can change subjective social norms. Therefore, cues such as pictures, likes, and comments can amplify the impact of social norm appeals on social media (cf. Dempsey et al., 2018). In addition, the reception of a video including social norm appeals could lead to recipients paying more attention to the target behavior. Consequently, more norm-confirming content could be recognized on social media, especially for topics new to the recipient. When people learn that more people are changing their behavior and can view this on their social media feed, it could enhance the internalization of social norms and foster behavioral change (cf. Lutkenhaus et al., 2023).

Furthermore, the effects of social norm appeals can be amplified by algorithms (Lutkenhaus et al., 2023). As people view content on social media that contains social norm appeals and engage further with the topic, it is likely that similar content will be suggested by recommender systems. Even if the suggested content does not contain other social norm appeals, perceived social norms can be reinforced when showing commitment to the target behavior or emphasizing the importance of the action by suggesting similar content (cf. Spartz et al., 2017; Lutkenhaus et al., 2023).

However, further research is required to examine long-term effects, for example, whether even a short-term change in perceived norms combined with algorithm-based suggestions can lead to amplified and reinforcing effects. Offline field experiments indicate that social norm appeals can develop their full effect over some time (e.g.,Kormos et al., 2015; Sparkman et al., 2021; Berger et al., 2022), but there are few studies in the context of environmental behavior on social media. Nevertheless, such research is highly relevant, on the one hand because individuals can be addressed repeatedly in ads on social media. On the other hand, the reference groups mentioned in social norm appeals can be addressed quite specifically, which can amplify the effects of social norm appeals (Bollinger et al., 2023; Chung and Lapinski, 2023; Lutkenhaus et al., 2023).

## 7 Limitations

One limitation of this study is that it was an online experiment and did not measure real-world environmental behavior. On the one hand, the intention to offset and the calculation of the price are easy to implement. On the other hand, the online setting can also be subject to researcher demand or desirability biases because it is no social situation (cf. Habib et al., 2021). Furthermore, as discussed earlier, this may have influenced the impact of the descriptive appeals and injunctive appeals on persuasive outcomes. In general, the results are limited because only a specific behavior (VCO) was examined in a specific medium (explainer video). What is particularly critical about VCO as a research topic is that there are apparently widespread reasons to speak out against it which can affect offset behavior and intentions (e.g., Choi et al., 2016; Schorn et al., 2023). This problem is also reflected in the comments at the end of our studies.

In addition, we used a broad sample representing the German population and formed groups based on environmental awareness or personal norms. This indicates that the reference group was “Germans” and therefore broad and the same for all groups. However, social norm appeals may be particularly effective for individuals who feel similar to the target group (Goldstein et al., 2008; Smith and Louis, 2009), and a dynamic descriptive appeal in particular may be more effective when the reference group is viewed as a similar group (Chung and Lapinski, 2023). Other studies that demonstrate the positive effects of social norm appeals use closer reference groups, such as students at the same university (Mortensen et al., 2019) or guests at the same cafeteria (Loschelder et al., 2019). Therefore, the reference group may have been too broad in this study. Furthermore, some studies indicating differences depending on personal characteristics have used different samples and reference groups (e.g., Demarque et al., 2015; Richter et al., 2018). Therefore, our results cannot exclude the possibility that the hypothesized results occurred under different circumstances. At the operational level, our results could be limited by the fact that we included the logo of our university in the questionnaire, but we conducted the study in another country, which could have affected the perception of the reported reference group (cf. Melnyk et al., 2019; Chung and Lapinski, 2023). Finally, the results of the first study may be limited by the COVID-19 pandemic, as air travel was restricted.

## 8 Conclusion

In summary, we found that social norm appeals influenced perceived social norms in both studies, but there was only an effect of the injunctive majority appeal on persuasive outcomes in the first study. In both studies, there were no differences between the descriptive conditions or differences depending on individuals’ environmental awareness or personal norms. It is possible that a single exposure was not enough to produce persuasive effects and that the change in perceived social norms had to be internalized first. In online explainer videos, the impact of social norm appeals could be enhanced by algorithm-based suggestions and when social norm appeals draw attention to norm-compliant content. However, further research is required on the long-term effects and possible amplifications of the effects on social media because social norm appeals would be relatively easy to implement as a measure to promote target behavior actively. Nevertheless, injunctive majority appeal could effectively communicate that the vast majority of people support climate change action and expect their national government to act (cf. Andre et al., 2024). For those who want to actively promote environmentally friendly minority behavior, emphasizing that a majority approves of the behavior appears to be an easy and low-risk way to go about it.

## Data availability statement

The datasets presented in this study can be found in online repositories. The names of the repository/repositories and accession number(s) can be found at: https://osf.io/87y6t/.

## Ethics statement

The studies involving humans were approved by Ethics Committee of the Faculty of Arts and Social Sciences (University of Zurich). The studies were conducted in accordance with the local legislation and institutional requirements. The participants provided their written informed consent to participate in this study.

## Author contributions

AS: Software, Writing – review & editing, Writing – original draft, Visualization, Project administration, Methodology, Investigation, Formal analysis, Data curation, Conceptualization. WW: Writing – review & editing, Conceptualization, Methodology, Supervision, Formal analysis, Methodology, Conceptualization.

## Footnotes

^1^ https://osf.io/87y6t/?view_only=bb647eae503446d5b03aa58e777214fe

^2^Please note that results of the analyses of variance, conducted without including covariates, are consistent with the reported findings.

## References

[ref1] Abrahamse W. Steg L. (2013). Social influence approaches to encourage resource conservation: a meta-analysis. Glob. Environ. Chang. 23, 1773–1785. doi: 10.1016/j.gloenvcha.2013.07.029

[ref2] Alblas M. C. Meijers M. H. C. De Groot H. E. Mollen S. (2023). “Meat” me in the middle: the potential of a social norm feedback intervention in the context of meat consumption – a conceptual replication. Environ. Commun. 17, 991–1003. doi: 10.1080/17524032.2022.2149587

[ref9003] Allcott H. (2011). Social norms and energy conservation. Journal of Public Economics, 95, 1082–1095. doi: 10.1016/j.jpubeco.2011.03.003

[ref3] Andre P. Boneva T. Chopra F. Falk A. (2024). Globally representative evidence on the actual and perceived support for climate action. Nat. Clim. Chang. 14, 253–259. doi: 10.1038/s41558-024-01925-3

[ref4] Aruta J. J. B. R. (2022). An extension of the theory of planned behaviour in predicting intention to reduce plastic use in the Philippines: cross-sectional and experimental evidence. Asian J. Soc. Psychol. 25, 406–420. doi: 10.1111/ajsp.12504

[ref5] Baiardi D. Morana C. (2021). Climate change awareness: empirical evidence for the European Union. Energy Econ. 96:105163. doi: 10.1016/j.eneco.2021.105163

[ref6] Bamberg S. Hunecke M. Blöbaum A. (2007). Social context, personal norms and the use of public transportation: two field studies. J. Environ. Psychol. 27, 190–203. doi: 10.1016/j.jenvp.2007.04.001

[ref7] Berger J. (2021). Social tipping interventions can promote the diffusion or decay of sustainable consumption norms in the field: evidence from a quasi-experimental intervention study. Sustain. For. 13:3529. doi: 10.3390/su13063529

[ref8] Berger S. Kilchenmann A. Lenz O. Schlöder F. (2022). Willingness-to-pay for carbon dioxide offsets: field evidence on revealed preferences in the aviation industry. Glob. Environ. Chang. 73:102470. doi: 10.1016/j.gloenvcha.2022.102470

[ref9] Bergquist M. Nilsson A. (2016). I saw the sign: promoting energy conservation via normative prompts. J. Environ. Psychol. 46, 23–31. doi: 10.1016/j.jenvp.2016.03.005

[ref9001] Bergquist M. Nilsson A. Schultz W. P. (2019). A meta-analysis of field-experiments using social norms to promote pro-environmental behaviors. Global Environmental Change, 59, 101941. doi: 10.1016/j.gloenvcha.2019.101941

[ref9002] Bhanot S. P. (2018). Isolating the effect of injunctive norms on conservation behavior: New evidence from a field experiment in California. Organizational Behavior and Human Decision Processes, S0749597818305636. doi: 10.1016/j.obhdp.2018.11.002

[ref10] Bollinger B. Gillingham K. T. Wight K. G. (2023). Making prosocial social: the effectiveness of social proof for energy conservation using social media. J. Assoc. Consum. Res. 8, 290–300. doi: 10.1086/725031

[ref9004] Boenke L. Panning M. Thurow A. Hörisch J. Loschelder D. D. (2022). Who can nudge for sustainable development? How nudge source renders dynamic norms (in-)effective in eliciting sustainable behavior. Journal of Cleaner Production, 368, 133246. doi: 10.1016/j.jclepro.2022.133246

[ref9005] Bonan J. Cattaneo C. d’Adda G. Tavoni M. (2020). The interaction of descriptive and injunctive social norms in promoting energy conservation. Nature Energy, 5, 900–909. doi: 10.1038/s41560-020-00719-z

[ref11] Boon-Falleur M. Grandin A. Baumard N. Chevallier C. (2022). Leveraging social cognition to promote effective climate change mitigation. Nat. Clim. Chang. 12, 332–338. doi: 10.1038/s41558-022-01312-w

[ref12] Boy B. Bucher H.-J. Christ K. (2020). Audiovisual science communication on tv and YouTube: how recipients understand and evaluate science videos. Front. Commun. 5:608620. doi: 10.3389/fcomm.2020.608620

[ref13] Brechin S. R. Bhandari M. (2011). Perceptions of climate change worldwide. WIREs Clim. Change 2, 871–885. doi: 10.1002/wcc.146

[ref14] Carfora V. Zeiske N. van der Werff E. Steg L. Catellani P. (2022). Adding dynamic norm to environmental information in messages promoting the reduction of meat consumption. Environ. Commun. 16, 900–919. doi: 10.1080/17524032.2022.2062019

[ref15] Carmichael M. Bender J. Chiarelli N. Clemence M. Ryan Ing B. (2023). Global trends2023: a new world disorder? Navigating a polycrisis. Ipsos. Available at: https://www.ipsos.com/sites/default/files/2023-Ipsos-Global-Trends-Report.pdf

[ref9006] Cialdini R. B. (2003). Crafting normative messages to protect the environment. Current Directions in Psychological Science, 12, 105–109. doi: 10.1111/1467-8721.01242

[ref16] Choi A. S. Ritchie B. W. Fielding K. S. (2016). A mediation model of air travelers’ voluntary climate action. J. Travel Res. 55, 709–723. doi: 10.1177/0047287515581377

[ref17] Chung M. Lapinski M. K. (2019). Extending the theory of normative social behavior to predict hand-washing among Koreans. Health Commun. 34, 1120–1129. doi: 10.1080/10410236.2018.1461586, PMID: 29634374

[ref18] Chung M. Lapinski M. K. (2023). The effect of dynamic norms messages and group identity on pro-environmental behaviors. Commun. Res. 51, 439–462. doi: 10.1177/00936502231176670

[ref19] Chung A. Rimal R. N. (2016). Social norms: a review. Rev. Commun. Res. 4, 1–28. doi: 10.12840/issn.2255-4165.2016.04.01.008

[ref20] Cialdini R. B. Jacobson R. P. (2021). Influences of social norms on climate change-related behaviors. Curr. Opin. Behav. Sci. 42, 1–8. doi: 10.1016/j.cobeha.2021.01.005

[ref21] Cialdini R. B. Kallgren C. A. Reno R. R. (1991). “A focus theory of normative conduct: a theoretical refinement and reevaluation of the role of norms in human behavior” in Advances in experimental social psychology, Ed. Mark P. Zanna (Academic Press), 24, 201–234.

[ref22] Cialdini R. B. Reno R. R. Kallgren C. A. (1990). A focus theory of normative conduct: recycling the concept of norms to reduce littering in public places. J. Pers. Soc. Psychol. 58, 1015–1026. doi: 10.1037/0022-3514.58.6.1015

[ref23] Dawes J. (2008). Do data characteristics change according to the number of scale points used? An experiment using 5-point, 7-point and 10-point scales. Int. J. Mark. Res. 50, 61–104. doi: 10.1177/147078530805000106

[ref24] de Groot J. I. M. Bondy K. Schuitema G. (2021). Listen to others or yourself? The role of personal norms on the effectiveness of social norm interventions to change pro-environmental behavior. J. Environ. Psychol. 78:101688. doi: 10.1016/j.jenvp.2021.101688

[ref25] de Groot J. I. M. Schuitema G. (2012). How to make the unpopular popular? Policy characteristics, social norms and the acceptability of environmental policies. Environ. Sci. Pol. 19-20, 100–107. doi: 10.1016/j.envsci.2012.03.004

[ref9008] DellaValle N. Zubaryeva A. (2019). Can we hope for a collective shift in electric vehicle adoption? Testing salience and norm-based interventions in South Tyrol, Italy. Energy Research & Social Science, 55, 46–61. doi: 10.1016/j.erss.2019.05.005

[ref26] Demarque C. Charalambides L. Hilton D. J. Waroquier L. (2015). Nudging sustainable consumption: the use of descriptive norms to promote a minority behavior in a realistic online shopping environment. J. Environ. Psychol. 43, 166–174. doi: 10.1016/j.jenvp.2015.06.008

[ref27] Dempsey R. C. McAlaney J. Bewick B. M. (2018). A critical appraisal of the social norms approach as an interventional strategy for health-related behavior and attitude change. Front. Psychol. 9:2180. doi: 10.3389/fpsyg.2018.02180, PMID: 30459694 PMC6232455

[ref28] Denton G. Chi O. H. Gursoy D. (2020). An examination of the gap between carbon offsetting attitudes and behaviors: role of knowledge, credibility and trust. Int. J. Hosp. Manag. 90:102608. doi: 10.1016/j.ijhm.2020.102608

[ref29] Economist Intelligence Unit (2021). An eco-wakening: measuring awareness, engagement, and action for nature. Available at: https://files.worldwildlife.org/wwfcmsprod/files/Publication/file/93ts5bhvyq_An_EcoWakening_Measuring_awareness__engagement_and_action_for_nature_FINAL_MAY_2021.pdf

[ref30] Elgaaied-Gambier L. Monnot E. Reniou F. (2018). Using descriptive norm appeals effectively to promote green behavior. J. Bus. Res. 82, 179–191. doi: 10.1016/j.jbusres.2017.09.032

[ref31] Eriksson K. Strimling P. Coultas J. C. (2015). Bidirectional associations between descriptive and injunctive norms. Organ. Behav. Hum. Decis. Process. 129, 59–69. doi: 10.1016/j.obhdp.2014.09.011

[ref32] Ettinger J. Walton P. Painter J. DiBlasi T. (2021). Climate of hope or doom and gloom? Testing the climate change hope vs. fear communications debate through online videos. Clim. Chang. 164:19. doi: 10.1007/s10584-021-02975-8

[ref33] European Commission (2020). Special Eurobarometer 501: attitudes of Europeans towards the environment. Available at: https://europa.eu/eurobarometer/surveys/detail/2257

[ref34] European Commission (2021). Special Eurobarometer 513: climate change. Available at: http://data.europa.eu/88u/dataset/s2273_95_1_513_eng

[ref36] Farrow K. Grolleau G. Ibanez L. (2017). Social norms and pro-environmental behavior: a review of the evidence. Ecol. Econ. 140, 1–13. doi: 10.1016/j.ecolecon.2017.04.017

[ref37] Fell H.-J. Traber T. (2020). The path to climate neutrality by 2050 misses the Paris climate targets: the rocky road to truthfulness in climate politics (EWG policy paper). Energy Watch Group. Available at: https://www.energywatchgroup.org/wp-content/uploads/EWG_Policy-Paper_2021_Climate-Neutrality-2050.pdf

[ref38] Finstad K. (2010). Response interpolation and scale sensitivity: evidence against 5-point scales. J. Usability Stud. 5, 104–110.

[ref9009] IPCC. (2021). Climate Change 2021: The Physical Science Basis. Contribution of Working Group I to the Sixth Assessment Report of the Intergovernmental Panel on Climate Change (6.). Cambridge University Press. https://www.ipcc.ch/report/ar6/wg1/#SPM

[ref39] Ge W. Sheng G. Zhang H. (2020). How to solve the social norm conflict dilemma of green consumption: the moderating effect of self-affirmation. Front. Psychol. 11:566571. doi: 10.3389/fpsyg.2020.566571, PMID: 33329200 PMC7732647

[ref40] Geiger S. Holzhauer B. (2020). Weiterentwicklung einer Skala zur Messung von zentralen Kenngrößen des Umweltbewusstseins (p. 74). Umweltbundesamt. Available at: https://www.umweltbundesamt.de/publikationen/weiterentwicklung-skala-umweltbewusstsein

[ref41] Göckeritz S. Schultz P. W. Rendón T. Cialdini R. B. Goldstein N. J. Griskevicius V. (2009). Descriptive normative beliefs and conservation behavior: the moderating roles of personal involvement and injunctive normative beliefs. Eur. J. Soc. Psychol. 40, 514–523. doi: 10.1002/ejsp.643

[ref42] Goldstein N. J. Cialdini R. B. Griskevicius V. (2008). A room with a viewpoint: using social norms to motivate environmental conservation in hotels. J. Consum. Res. 35, 472–482. doi: 10.1086/586910

[ref43] Gossen M. Tröger J. Veneny M. Eichhorn H. Bergener J. (2023). Do people make sufficiency-oriented mobile phone choices based on dynamic norms? The perception and effectiveness of sufficiency-promoting messages in online media. Front. Sustain. 4:1145243. doi: 10.3389/frsus.2023.1145243

[ref44] Gössling S. Haglund L. Kallgren H. Revahl M. Hultman J. (2009). Swedish air travellers and voluntary carbon offsets: towards the co-creation of environmental value? Curr. Issue Tour. 12, 1–19. doi: 10.1080/13683500802220687

[ref45] Habib R. White K. Hoegg J. (2021). Everybody thinks we should but nobody does: how combined injunctive and descriptive norms motivate organ donor registration. J. Consum. Psychol. 31, 621–630. doi: 10.1002/jcpy.1220

[ref46] Hayes A. F. (2018). Introduction to mediation, moderation, and conditional process analysis: a regression-based approach. 2nd Edn. New York: The Guilford Press.

[ref9007] He H. Fu J. Li X. Guo R. (2019). The interplay between endorser social status and normative appeals on the endorsement effectiveness of pro-environmental behaviors. PLOS ONE, 14, e0210699. doi: 10.1371/journal.pone.0210699 30645643 PMC6333400

[ref47] Hertwich E. G. Peters G. P. (2009). Carbon footprint of nations: a global, trade-linked analysis. Environ. Sci. Technol. 43, 6414–6420. doi: 10.1021/es803496a, PMID: 19746745

[ref48] Jacobson R. P. Marchiondo L. A. Jacobson K. J. L. Hood J. N. (2020). The synergistic effect of descriptive and injunctive norm perceptions on counterproductive work behaviors. J. Bus. Ethics 162, 191–209. doi: 10.1007/s10551-018-3968-1

[ref49] Jacobson R. P. Mortensen C. R. Cialdini R. B. (2011). Bodies obliged and unbound: differentiated response tendencies for injunctive and descriptive social norms. J. Pers. Soc. Psychol. 100, 433–448. doi: 10.1037/a0021470, PMID: 21171790

[ref50] Kácha O. van der Linden S. (2021). The moderating role of moral norms and personal cost in compliance with pro-environmental social norms. Curr. Res. Ecol. Soc. Psychol. 2:100020. doi: 10.1016/j.cresp.2021.100020

[ref51] Kane J. V. (2024). More than meets the ITT: a guide for anticipating and investigating nonsignificant results in survey experiments. J. Exp. Polit. Sci. 1–16, 1–16. doi: 10.1017/XPS.2024.1

[ref52] Kim S. H. Seock Y.-K. (2019). The roles of values and social norm on personal norms and pro-environmentally friendly apparel product purchasing behavior: the mediating role of personal norms. J. Retail. Consum. Serv. 51, 83–90. doi: 10.1016/j.jretconser.2019.05.023

[ref53] Koch W. Bleisch N. (2020). Ergebnisse der ARD/ZDF-Onlinestudie 2020: Erneut starke Zuwächse bei Onlinevideo. Media Perspektiven 9, 482–500.

[ref54] Kormos C. Gifford R. Brown E. (2015). The influence of descriptive social norm information on sustainable transportation behavior: a field experiment. Environ. Behav. 47, 479–501. doi: 10.1177/0013916513520416

[ref55] Lalot F. Falomir-Pichastor J. M. Quiamzade A. (2018). Compensation and consistency effects in proenvironmental behaviour: the moderating role of majority and minority support for proenvironmental values. Group Process. Intergroup Relat. 21, 403–421. doi: 10.1177/1368430217733117

[ref56] Lee S. J. Liu J. (2023). Leveraging dynamic norm messages to promote counter-normative health behaviors: the moderating role of current and future injunctive norms, attitude and self-efficacy. Health Commun. 38, 1071–1079. doi: 10.1080/10410236.2021.1991638, PMID: 34689673

[ref57] Lee T. M. Markowitz E. M. Howe P. D. Ko C.-Y. Leiserowitz A. A. (2015). Predictors of public climate change awareness and risk perception around the world. Nat. Clim. Chang. 5, 1014–1020. doi: 10.1038/nclimate2728

[ref58] Loschelder D. D. Siepelmeyer H. Fischer D. Rubel J. A. (2019). Dynamic norms drive sustainable consumption: norm-based nudging helps café customers to avoid disposable to-go-cups. J. Econ. Psychol. 75:102146. doi: 10.1016/j.joep.2019.02.002

[ref59] Lu J.-L. Wang C.-Y. (2018). Investigating the impacts of air travellers’ environmental knowledge on attitudes toward carbon offsetting and willingness to mitigate the environmental impacts of aviation. Transp. Res. Part D: Transp. Environ. 59, 96–107. doi: 10.1016/j.trd.2017.12.024

[ref60] Lutkenhaus R. O. McLarnon C. Walker F. (2023). Norms-shifting on social media: a review of strategies to shift health-related norms among adolescents and young adults on social media. Rev. Commun. Res. 11, 127–149. doi: 10.5680/RCR.V11.5

[ref61] Mair J. (2011). Exploring air travellers’ voluntary carbon-offsetting behaviour. J. Sustain. Tour. 19, 215–230. doi: 10.1080/09669582.2010.517317

[ref62] Matthes J. Marquart F. Naderer B. Arendt F. Schmuck D. Adam K. (2015). Questionable research practices in experimental communication research: a systematic analysis from 1980 to 2013. Commun. Methods Meas. 9, 193–207. doi: 10.1080/19312458.2015.1096334

[ref63] Melnyk V. van Herpen E. Jak S. van Trijp H. C. M. (2019). The mechanisms of social norms’ influence on consumer cecision making: a meta-analysis. Z. Psychol. 227, 4–17. doi: 10.1027/2151-2604/a000352

[ref9010] McDonald R. I. Fielding K. S. Louis W. R. (2014a). Conflicting norms highlight the need for action. Environment and Behavior, 46, 139–162. doi: 10.1177/0013916512453992

[ref9011] McDonald R. I. Fielding K. S. Louis W. R. (2014b). Conflicting social norms and community conservation compliance. Journal for Nature Conservation, 22, 212–216. doi: 10.1016/j.jnc.2013.11.005

[ref64] Meyer A. (2015). Does education increase pro-environmental behavior? Evidence from Europe. Ecol. Econ. 116, 108–121. doi: 10.1016/j.ecolecon.2015.04.018

[ref65] Miller D. T. Prentice D. A. (1996). The construction of social norms and standards. In Social psychology: Handbook of basic principles, Eds. E. T. Higgins and A. W. Kruglanski. (New York: The Guilford Press), 799–829.

[ref66] Mortensen C. R. Neel R. Cialdini R. B. Jaeger C. M. Jacobson R. P. Ringel M. M. (2019). Trending norms: a lever for encouraging behaviors performed by the minority. Soc. Psychol. Personal. Sci. 10, 201–210. doi: 10.1177/1948550617734615

[ref67] Moscovici S. Personnaz B. (1980). Studies in social influence. J. Exp. Soc. Psychol. 16, 270–282. doi: 10.1016/0022-1031(80)90070-0

[ref68] Nolan J. M. (2021). Social norm interventions as a tool for pro-climate change. Curr. Opin. Psychol. 42, 120–125. doi: 10.1016/j.copsyc.2021.06.001, PMID: 34280794

[ref69] Oliver M. C. Adkins M. J. (2020). “Hot-headed” students? Scientific literacy, perceptions and awareness of climate change in 15-year olds across 54 countries. Energy Res. Soc. Sci. 70:101641. doi: 10.1016/j.erss.2020.101641

[ref70] Onwezen M. C. Antonides G. Bartels J. (2013). The norm activation model: an exploration of the functions of anticipated pride and guilt in pro-environmental behaviour. J. Econ. Psychol. 39, 141–153. doi: 10.1016/j.joep.2013.07.005

[ref71] Passafaro P. (2020). Attitudes and tourists’ sustainable behavior: an overview of the literature and discussion of some theoretical and methodological issues. J. Travel Res. 59, 579–601. doi: 10.1177/0047287519851171

[ref72] Poškus M. S. (2018). Investigating pro-environmental behaviors of Lithuanian university students. Curr. Psychol. 37, 225–233. doi: 10.1007/s12144-016-9506-3

[ref74] Reno R. R. Cialdini R. B. Kallgren C. A. (1993). The transsituational influence of social norms. J. Pers. Soc. Psychol. 64, 104–112. doi: 10.1037/0022-3514.64.1.104

[ref75] Rhodes N. Shulman H. C. McClaran N. (2020). Changing norms: a meta-analytic integration of research on social norms appeals. Hum. Commun. Res. 46, 161–191. doi: 10.1093/hcr/hqz023

[ref76] Richter I. Thøgersen J. Klöckner C. (2018). A social norms intervention going wrong: boomerang effects from descriptive norms information. Sustain. For. 10:2848. doi: 10.3390/su10082848

[ref77] Ritchie B. W. Kemperman A. Dolnicar S. (2021). Which types of product attributes lead to aviation voluntary carbon offsetting among air passengers? Tour. Manag. 85:104276. doi: 10.1016/j.tourman.2020.104276

[ref79] Schorn A. (2022). Online explainer videos: features, benefits, and effects. Front. Commun. 7:1034199. doi: 10.3389/fcomm.2022.1034199

[ref80] Schorn A. Schläpfer S. Wirth W. (2023). “Promoting voluntary carbon offsetting through social norm appeals: some learnings from null results” in Klima(wandel)kommunikation: Im Spannungsfeld von Wissenschaft, Medien und öffentlicher Meinung. eds. Wolling J. Becker M. Schumann C., vol. 8. 1st ed (Ilmenau: Universitätsverlag Ilmenau), 207–224.

[ref81] Schorn A. Wirth W. (2023). Meet bob and offset your flight: optimising explainer videos to promote voluntary carbon offsetting. Media Commun. 11, 349–360. doi: 10.17645/mac.v11i1.6028

[ref82] Schultz P. W. Khazian A. M. Zaleski A. C. (2008). Using normative social influence to promote conservation among hotel guests. Soc. Influ. 3, 4–23. doi: 10.1080/15534510701755614

[ref83] Schultz P. W. Messina A. Tronu G. Limas E. F. Gupta R. Estrada M. (2016). Personalized normative feedback and the moderating role of personal norms: a field experiment to reduce residential water consumption. Environ. Behav. 48, 686–710. doi: 10.1177/0013916514553835

[ref84] Schultz P. W. Nolan J. M. Cialdini R. B. Goldstein N. J. Griskevicius V. (2007). The constructive, destructive, and reconstructive power of social norms. Psychol. Sci. 18, 429–434. doi: 10.1111/j.1467-9280.2007.01917.x, PMID: 17576283

[ref85] Seger B. T. Burkhardt J. Straub F. Scherz S. Nieding G. (2023). Reducing the individual carbon impact of video streaming: a seven-week intervention using information, goal setting, and feedback. J. Consum. Policy 46, 137–153. doi: 10.1007/s10603-023-09536-9, PMID: 36815974 PMC9923665

[ref86] Segerstedt A. Grote U. (2016). Increasing adoption of voluntary carbon offsets among tourists. J. Sustain. Tour. 24, 1541–1554. doi: 10.1080/09669582.2015.1125357

[ref9012] Shealy T. Johnson E. Weber E. Klotz L. Applegate S. Ismael D.. (2018). Providing descriptive norms during engineering design can encourage more sustainable infrastructure. Sustainable Cities and Society, 40, 182–188. doi: 10.1016/j.scs.2018.04.017

[ref9013] Shulman H. Rhodes N. Davidson E. Ralston R. Borghetti L. Morr L. (2017). The state of the field of social norms research. International Journal of Communication, 11. https://ijoc.org/index.php/ijoc/article/view/6055

[ref87] Smith J. R. Louis W. R. (2008). Do as we say and as we do: the interplay of descriptive and injunctive group norms in the attitude-behaviour relationship. Br. J. Soc. Psychol. 47, 647–666. doi: 10.1348/014466607X269748 18163950

[ref88] Smith J. R. Louis W. R. (2009). Group norms and the attitude-behaviour relationship. Soc. Personal. Psychol. Compass 3, 19–35. doi: 10.1111/j.1751-9004.2008.00161.x

[ref89] Smith J. R. Louis W. R. Terry D. J. Greenaway K. H. Clarke M. R. Cheng X. (2012). Congruent or conflicted? The impact of injunctive and descriptive norms on environmental intentions. J. Environ. Psychol. 32, 353–361. doi: 10.1016/j.jenvp.2012.06.001

[ref9014] Sparkman G. Weitz E. Robinson T. N. Malhotra N. Walton G. M. (2020). Developing a scalable dynamic norm menu-based intervention to reduce meat consumption. Sustainability, 12, 2453. doi: 10.3390/su12062453

[ref90] Sparkman G. Macdonald B. N. J. Caldwell K. D. Kateman B. Boese G. D. (2021). Cut back or give it up? The effectiveness of reduce and eliminate appeals and dynamic norm messaging to curb meat consumption. J. Environ. Psychol. 75:101592. doi: 10.1016/j.jenvp.2021.101592

[ref91] Sparkman G. Walton G. M. (2017). Dynamic norms promote sustainable behavior, even if it is counternormative. Psychol. Sci. 28, 1663–1674. doi: 10.1177/0956797617719950, PMID: 28961062

[ref92] Spartz J. T. Su L. Y.-F. Griffin R. Brossard D. Dunwoody S. (2017). YouTube, social norms and perceived salience of climate change in the American mind. Environ. Commun. 11, 1–16. doi: 10.1080/17524032.2015.1047887

[ref93] Streck C. (2021). How voluntary carbon markets can drive climate ambition. J. Energy Nat. Resour. Law 39, 367–374. doi: 10.1080/02646811.2021.1881275

[ref94] Thøgersen J. (2006). Norms for environmentally responsible behaviour: an extended taxonomy. J. Environ. Psychol. 26, 247–261. doi: 10.1016/j.jenvp.2006.09.004

[ref95] Thøgersen J. (2008). Social norms and cooperation in real-life social dilemmas. J. Econ. Psychol. 29, 458–472. doi: 10.1016/j.joep.2007.12.004

[ref96] Tversky A. Kahneman D. (1974). Judgment under uncertainty: heuristics and biases. Science, 185, 1124–1131. JSTOR. 17835457 10.1126/science.185.4157.1124

[ref97] United Nations. (2015). The sustainable development goals. 17 goals to transform our world. Available at: https://www.un.org/sustainabledevelopment/

[ref98] Van Valkengoed A. M. Steg L. (2019). Meta-analyses of factors motivating climate change adaptation behaviour. Nat. Clim. Chang. 9, 158–163. doi: 10.1038/s41558-018-0371-y

[ref99] Verkooijen K. T. Stok F. M. Mollen S. (2015). The power of regression to the mean: a social norm study revisited. Eur. J. Soc. Psychol. 45, 417–425. doi: 10.1002/ejsp.2111

[ref100] Vicente-Molina M. A. Fernández-Sainz A. Izagirre-Olaizola J. (2018). Does gender make a difference in pro-environmental behavior? The case of the Basque Country university students. J. Clean. Prod. 176, 89–98. doi: 10.1016/j.jclepro.2017.12.079

[ref101] Wang Y. Hao F. Liu Y. (2021). Pro-environmental behavior in an aging world: evidence from 31 countries. Int. J. Environ. Res. Public Health 18:1748. doi: 10.3390/ijerph18041748, PMID: 33670167 PMC7916887

[ref102] Welbourne D. J. Grant W. J. (2015). Science communication on YouTube: factors that affect channel and video popularity. Public Underst. Sci. 25, 706–718. doi: 10.1177/0963662515572068, PMID: 25698225

[ref103] Witzling L. Shaw B. Trechter D. (2019). Which communication channels shape normative perceptions about buying local food? An application of social exposure. Agric. Hum. Values 36, 443–454. doi: 10.1007/s10460-019-09926-1

[ref104] Wolf I. Ebersbach B. Huttarsch J.-H. (2023). Soziales Nachhaltigkeitsbarometer der Energie- und Verkehrswende 2023 (Soziales Nachhaltigkeitsbarometer). Potsdam-Institut für Klimafolgenforschung (PIK). Available at: https://snb.ariadneprojekt.de/soziales-nachhaltigkeitsbarometer

[ref105] Wulfsberg I. Reiser D. Rundshagen V. Scherle N. (2016). The influence of environmental attitudes and concerns on the voluntary carbon-offsetting behaviour of German tourists (BEST EN think tank XVI corporate responsibility in tourism – standards practices and policies): Building Excellence in Sustainable Tourism Education Network (Potsdam: BEST EN).

[ref106] Young S. D. Jordan A. H. (2013). The influence of social networking photos on social norms and sexual health behaviors. Cyberpsychol. Behav. Soc. Netw. 16, 243–247. doi: 10.1089/cyber.2012.0080, PMID: 23438268 PMC3624629

