# Supplementary material for: They approve but they don’t act: promoting sustainable minority behavior with (conflicting) social norm appeals
Source: Front Psychol. 2024 Jun 26;15:1337585. doi: 10.3389/fpsyg.2024.1337585 (PMC11233761; doi:10.3389/fpsyg.2024.1337585)
Supplement: Supplementary file 1 [file Data_Sheet_1.PDF]

Supplementary Material

STUDY 1 2
Stimulus material and storyboard 2
Measures 6
Overview of experimental groups and duration of videos 10

STUDY 2 11
Stimulus material and storyboard 11
Measures 18

STUDY 1

Stimulus material and storyboard

Scene & duration	Script	Visualisation
**Introduction**<br><br>18 Seconds	Das ist der eindrückliche Eisstrom des Aletschgletschers, der sich in der Schweiz befindet. Er ist der grösste und längste Gletscher in den Alpen. Doch seit 1850 ist er zurückgegangen. Grund dafür ist der menschengemachte Klimawandel. Die Situation ist alarmierend.<br><br>*This is the impressive ice stream of the Aletsch glacier, located in Switzerland. It is the largest and longest glacier in the Alps, but it has been receding since 1850. The reason for this is anthropogenic climate change. The situation is alarming.*	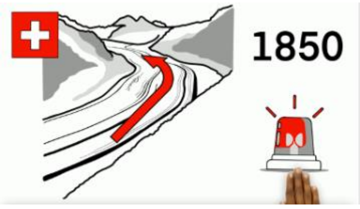
**Problem 1/4**<br><br>16 Seconds	Wir alle tragen mit unserem CO_2-Ausstoss dazu bei. Durchs Autofahren, Fliegen und Heizen werden Treibstoffe verbrannt und stossen so schädliche Gase aus. Diese gelangen dann in unsere Atmosphäre und verändern deren Zusammensetzung.<br><br>*We all contribute to this with our CO2 emissions. Driving, flying and heating burn fuels and emit harmful gases. These then get into our atmosphere and change its structure.*	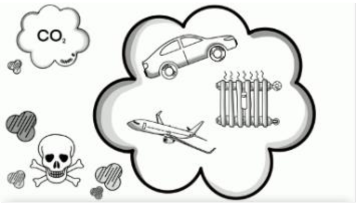
**Problem 2/4**<br><br>11 Seconds	Die Folge davon ist, dass mehr Sonnenstrahlen als bisher auf die Erde reflektiert werden. Wie im Treibhaus erwärmt sich dadurch die Luft auf der Erde deutlich mehr als normal.<br><br>*The result is that more of the sun's rays are reflected onto the earth than before. As in a greenhouse, this causes the air on Earth to heat up significantly more than normal.*	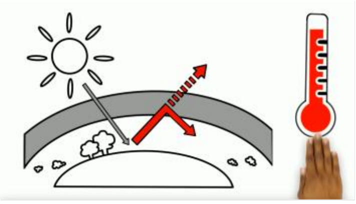

Problem 3/4

9 Seconds

Diese Erwärmung hat direkt spürbare Auswirkungen wie zum Beispiel Hitzewellen, Schmelzen der Gletscher oder heftige Regenfälle.

This warming has directly tangible effects such as heat waves, melting glaciers or heavy rainfall.

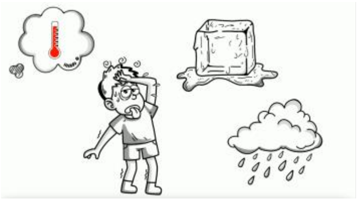

Problem 4/4

15 Seconds

Wir alle sind für diese extremen Ereignisse mitverantwortlich. Denn im Durchschnitt verursacht die deutsche Bevölkerung pro Person 11 Tonnen CO_2 pro Jahr. Würden auf der ganzen Welt alle so verschwenderisch leben, bräuchten wir 3 Erden.

We are all partly responsible for these extreme events. On average, the German population produces 11 tonnes of CO2 per person per year. If everyone in the world lived so lavishly, we would need 3 earths.

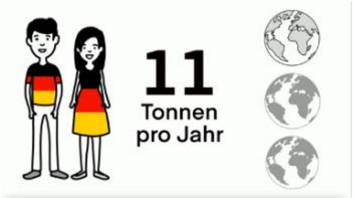

Solution 1/3

14 seconds

Die gute Nachricht: Du kannst deinen eigenen CO_2-Ausstoss kompensieren. Dies ist besonders sinnvoll bei Emissionen, die noch nicht vermeidbar sind. Zum Beispiel wenn du nicht auf einen Flug verzichten möchtest.

The good news is that you can offset your own CO2 emissions. This is particularly useful for emissions that cannot yet be avoided. For example, if you don't want to avoid a flight.

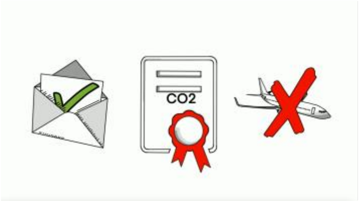

Solution 2/3

12 Seconds

Dafür musst du nicht einmal tief in die Tasche greifen. Du kannst deinen Flug von Berlin nach Mallorca für 17 Euro oder einen Flug nach Bali für 100 Euro kompensieren.

It's not even that expensive. You can offset your flight from Berlin to Mallorca for 17 euros or a flight to Bali for 100 euros.

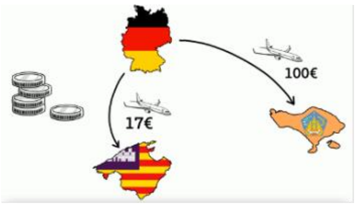

Solution 3/3

9 Seconds

Dieses Geld fliesst dann zum Beispiel in eine nachhaltige Waldbewirtschaftung in Deutschland oder in die Förderung von Solarenergie in Tansania.

This money then is invested, for example, in sustainable forest management in Germany or in the support of solar energy in Tanzania.

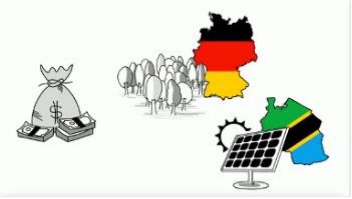

Explanation 1/2

9 seconds

Damit wird die durch den Flug ausgestossene Menge an CO2 an einer anderen Stelle auf der Erde eingespart und neutralisiert.

In this way, the amount of CO2 emitted by the flight is saved and neutralised elsewhere on earth.

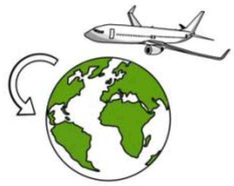

Explanation 2/2

17 seconds

Ärmere Länder profitieren dabei besonders, denn durch Klimaschutzprojekte wird auch Entwicklungsarbeit geleistet: CO2-Kompensationen helfen also dem Klima und gleichzeitig der lokalen Bevölkerung. Dadurch kann der menschengemachte Klimawandel verlangsamt werden.

Poorer countries benefit particularly, because climate protection projects also contribute to development: CO2 offsets therefore help the climate and the local population at the same time. In this way, anthropogenic climate change can be mitigated.

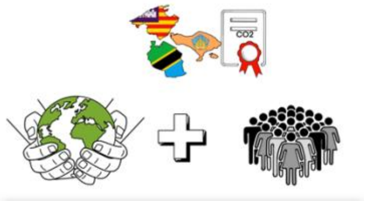

Static DSNA

13 Seconds

Doch wie viel wird in Deutschland kompensiert? Ein Blick auf die deutsche Bevölkerung zeigt, dass im Jahr 2021 nur eine von zehn Personen freiwillig ihren Flug kompensiert hat.

But how much is offset in Germany? A look at the German population demonstrates that in 2021 only one in ten people voluntarily offset their flight.

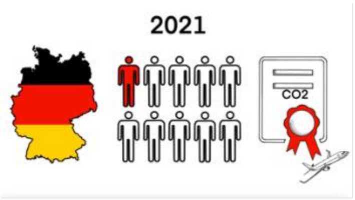

**Dynamic DSNA**<br><br>20 Seconds	Doch wie viel wird in Deutschland kompensiert? Bereits eine von zehn Personen hat im Jahr 2021 freiwillig ihren Flug kompensiert. Das sind fünf Mal mehr Personen als im Vorjahr. Es zeigt sich also ein positiver Trend und für die nächsten Jahre wird erwartet, dass sich der Anteil vervierfacht.<br><br>*But how much is offset in Germany? Already one in ten people voluntarily offset their flight in 2021. That is five times more people than in the previous year. So there is a positive trend and the proportion is expected to quadruple in the next few years.*	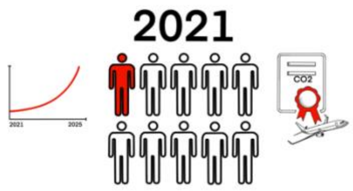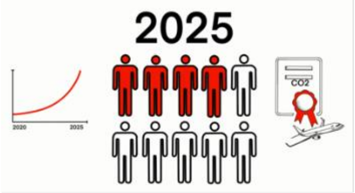
**ISNA**<br><br>20 seconds	Gleichzeitig zeigen viele Umfragen, dass eine deutliche Mehrheit der Deutschen Kompensationen befürwortet: bis zu 80% geben an, dass freiwillige CO2-Kompensationen im Flugverkehr sinnvoll, gut und wichtig sind.<br><br>*At the same time, many surveys show that a clear majority of Germans are in favour of offsets: up to 80% say that voluntary CO2 offsets in aviation are reasonable, good and important.*	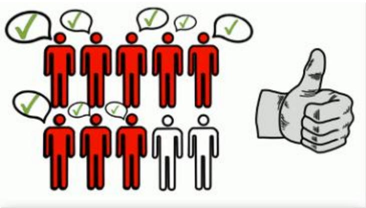
**ISNA (when there is no DSNA)**<br><br>20 seconds	Doch was halten die Deutschen von Kompensationen? Viele Umfragen zeigen, dass eine deutliche Mehrheit der Deutschen Kompensationen befürwortet: bis zu 80% geben an, dass freiwillige CO2-Kompensationen im Flugverkehr sinnvoll, gut und wichtig sind.<br><br>*But what do Germans think of offsets? Many surveys show that a clear majority of Germans are in favour of offsets: up to 80% say that voluntary CO2 offsets in aviation are reasonable, good and important.*	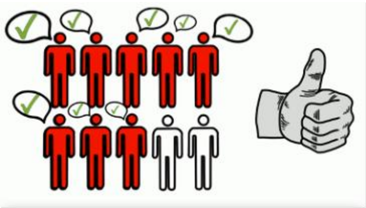

**End**<br><br>17 Seconds	Freiwillige CO_2-Kompensationen im Flugverkehr sind eine wichtige Lösung, um unseren Lebensraum und das Klima zu schützen. Gemeinsam sorgen wir so dafür, dass auch noch unsere Kinder die spektakulären Gletscher bestaunen können.<br><br>*Voluntary CO2 offsets for air travel are an important solution to protect our biosphere and the climate. Together, we can ensure that our children will still be able to marvel at the spectacular glaciers.*	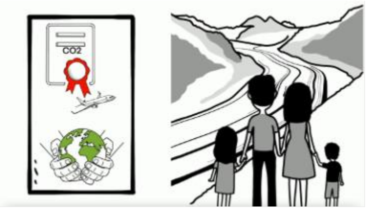

Note. The storyboard was translated with AI (deepl.com).

Measures

Variable	Statement (GER)	Source
Intention to offset	Ich erwäge es, in Zukunft CO2-Kompensationen für meine Flüge zu leisten. (*I am considering making carbon offsets for my flights in the future*.)<br><br>Ich gehe davon aus, dass ich in Zukunft CO2-Kompensationen für meine Flüge leisten werde. (*I expect to make carbon offsets for my flights in the future*.)<br><br>Ich möchte in Zukunft auf jeden Fall CO2-Kompensationen für meine Flüge leisten. (*I would definitely like to make carbon offsets for my flights in the future*.)<br><br>Ich bin motiviert, CO2-Kompensationen für meine Flüge zu leisten. (*I am motivated to offset emissions for my flights*.)<br><br>Ich habe vor, CO2-Kompensationen für meine Flüge zu leisten. (*I plan to offset the emissions from my flights*.)	following Denton et al. (2020)
Attitude towards VCO	Ich finde, dass freiwillige CO2-Kompensationen eine gute Sache sind. (*I think voluntary carbon offsetting is a good thing*.)<br><br>Eine freiwillige CO2-Kompensation ist sinnvoll. (*Voluntary carbon offsetting is useful*.)<br><br>Ich befürworte freiwillige CO2-Kompensationen. (*I approve of voluntary carbon offsets.*)	following Denton et al. (2020)
Perceived effectiveness of VCO	Freiwillige CO2-Kompensationen ermöglichen eine bessere Welt für mich und meine Kinder. (*Voluntary carbon offsets will provide a better world for me and my children*)<br><br>Freiwillige CO2-Kompensationen helfen Menschen zu einer besseren Lebensqualität. (*Voluntary carbon offsets will help people have a better quality of life.*)<br><br>Freiwillige CO2-Kompensationen tragen zur Reduktion des weltweiten CO2-Ausstoßes bei. (*Voluntary carbon offsets will reduce worldwide carbon emissions.*)	following Choi et al. (2016) and self-created items

	Freiwillige CO2-Kompensationen bremsen den vom Menschen verursachten Klimawandel. (*Voluntary carbon offsets slow down man-made climate change*.)	
	Freiwillige CO2-Kompensationen sind hilfreich, um den CO2-Ausstoß zu neutralisieren. (*Voluntary CO2 offsets are helpful in neutralizing CO2 emissions*.)	
	Ich bin der Meinung, dass man mit CO2-Kompensationen dem Klimawandel entgegenwirken kann. (*I believe that CO2 offsets can be used to counteract climate change*.)	
Perceived quality of the video	Wie hat das Video auf Sie gewirkt? Bitte denken Sie zurück an das Video und stufen Sie jeweils zwischen den zwei Gegensätzen ab, wie Sie das Video einschätzen. Das Video war ...<br>(*How did the video appear to you? Please think back to the video and grade how you rate the video between each of the two opposites. The video was ...*)	
	nicht überzeugend – überzeugend (*unconvincing – convincing*)	
	unverständlich – verständlich (*incomprehensible – understandable*)	
	unglaubwürdig – glaubwürdig (*unbelievable – credible*)	
	schlecht gemacht – gut gemacht (*badly done – well done*)	
	nicht vertrauenswürdig – vertrauenswürdig (*untrustworthy – trustworthy*)	
	nicht professionell – professionell (*not professional – professional*)	
Environmental awareness	Ich freue mich über Initiativen, die nachhaltige Lebensweisen einfach ausprobieren (z.B. Ökodörfer, SlowFood-Bewegung). (*I am happy about initiatives that simply try out sustainable ways of living (e.g., eco-villages, slow food movement).*)	Following Geiger and Holzhauer (2020)
	Es macht mich wütend, wenn ich sehe wie Deutschlang seine Klimaschutzziele verfehlt. (*It makes me angry when I see how Germany is missing its climate protection targets*.)	
	Die Umweltproblematik wird von vielen Umweltschützern stark übertrieben. (*The environmental problem is greatly exaggerated by many environmentalists*.) – r	
	Es beunruhigt mich, wenn ich daran denke, in welchen Umweltverhältnissen zukünftige Generationen wahrscheinlich leben müssen. (*It worries me when I think about the environmental conditions in which future generations will probably have to live*.)	
	Der Klimawandel bedroht auch die Lebensgrundlagen hier in Deutschland. (*Climate change also threatens the basis of life here in Germany*.)	
	Menschengemachte Umweltprobleme wie die Abholzung der Wälder oder auch das Plastik in den Weltmeeren empören mich. (*Man-made environmental problems such as deforestation or plastic in the world's oceans outrage me*.)	

	Es gibt natürliche Grenzen des Wachstums, die unsere industrialisierte Welt längst erreicht hat. (*There are natural limits to growth that our industrialised world has long since reached.*)	
	Jeder einzelne trägt Verantwortung dafür, dass wir nachfolgenden Generationen eine lebenswerte Umwelt hinterlassen. (*Each and every one of us is responsible for leaving a liveable environment for future generations*.)	
	Mehr Umweltschutz bedeutet auch mehr Lebensqualität und Gesundheit für alle. (*More environmental protection also means more quality of life and health for all*.)	
	Zugunsten der Umwelt sollten wir alle bereits sein, unseren derzeitigen Lebensstandard einzuschränken. (*For the sake of the environment, we should all be prepared to reduce our current standard of living*.)	
	Wir sollten nicht mehr Ressourcen verbrauchen als nachwachsen können. (*We should not consume more resources than can grow back*.)	
	Wir müssen Wege finden, wie wir unabhängig vom Wirtschaftswachstum gut leben können. (*We need to find ways to live well independently of economic growth*.)	
	Ich kaufe Lebensmittel aus kontrolliert-biologischem Anbau. (*I buy food from controlled organic cultivation*.)	
	Beim Einkaufen wähle ich Produkte mit Umweltsiegel (z.B. blauer Engel, EU Biosiegel oder EU Ecolabel). (*When I go shopping, I choose products with an environmental label (e.g. Blue Angel, EU Biosiegel or EU Ecolabel).*)	
	Ich spende Geld für Umwelt- und Naturschutzgruppen. (*I donate money to environmental and nature conservation groups*.) – dropped due to lack of reliability	
Perceived descriptive social norms	Was denken Sie: Wie groß schätzen Sie den Anteil derjenigen in Deutschland, die aktuell ihre Flüge freiwillig kompensieren? Bitte geben Sie eine Zahl zwischen 0 und 100 ein. [Der Anteil liegt bei … Prozent]<br>(*What do you think: How large do you estimate the proportion of those in Germany who currently compensate their flights voluntarily? Please enter a number between 0 and 100. [Percent]*)	cf. Mortensen et al. (2019); Sparkman and Walton (2017)
Perceived future descriptive norms (preconformity)	Was denken Sie: Wie stark wird der Anteil an Menschen, die ihre Flugreisen kompensieren, bis zum Jahr 2025 verändern? (´´)<br>Share will fall significantly (1) ← Share will remain unchanged (4) → Share will rise significantly (7)	cf. Sparkman and Walton (2017)
Perceived injunctive social norms	Mal abgesehen davon, wie viele Deutsche tatsächlich ihren Flug kompensieren: Was denken Sie, wie viel Prozent der Deutschen die freiwillige CO_2-Kompensation von Flugreisen befürworten? Bitte geben Sie eine Zahl zwischen 0 und 100 ein. In Deutschland befürworten …freiwillige CO2-	

	Kompensationen. Bitte geben Sie eine Zahl zwischen 0 und 100 ein. [Der Anteil liegt bei … Prozent]<br>(*Leaving aside how many Germans actually offset their flights: What percentage of Germans do you think support voluntary CO2 offsetting of air travel? Please enter a number between 0 and 100. [Percent]*))
Socio-demographics	Age, gender, education

Note. For all statements (except for socio-demographics), respondents could indicate their agreement from 1 = “strongly disagree” to 5 = “strongly agree”. The Items were translated with AI (deepl.com).

Overview of experimental groups and duration of videos

Experimental group	DSNA	ISNA	Duration
EG1	static	present	2 minutes 59 seconds
EG2	static	absent	2 minutes 43 seconds
EG3	dynamic	present	3 minutes 6 seconds
EG4	dynamic	absent	2 minutes 50 seconds
EG5	absent	present	2 minutes 48 seconds
EG6	absent	absent	2 minutes 27 seconds

STUDY 2

Stimulus material and storyboard

Please note that the English translation is an AI translation (deepl.com). They study was conducted in German.

Condition	Title	Length
Dynamic / ISNA:	Mehrheit befürwortet Kompensationen: Warum immer mehr ihre Flugreisen kompensieren<br>*Majority favors compensation: Why more and more are offsetting their air travel*	03:47
Dynamic / no ISNA:	Warum kompensieren immer mehr Menschen ihre Flugreisen?<br>*Why are more and more people offsetting their air travel?*	03:30
Static / no ISNA:	Warum erst wenige Menschen ihre Flugreisen kompensieren<br>*Why only a few people compensate for their air travel*	03:24
Static / ISNA:	Mehrheit befürwortet Kompensationen: Warum erst wenige Menschen ihre Flugreisen kompensieren<br>*Majority favors compensation: Why only a few people compensate for their air travel*	03:46
No DSNA / ISNA:	Warum die Mehrheit Kompensationen befürwortet<br>*Why the majority favors carbon offsets*	03:27
No DSNA / No ISNA:	Wie wirken sich Flugreisen auf die Umwelt aus?<br>*How does air travel affect the environment?*	02:59
PN:	Kompensationen: Wichtig und sinnvoll zum Klimaschutz<br>*Compensations: Important and useful for climate protection*	03:23

Note. Explainer videos can be found here:
https://osf.io/87y6t/?view_only=bb647eae503446d5b03aa58e777214fe

**Scene & duration**	**Script**	**Visualisation**
	**Constant in all videos**	
**Introduction**<br><br>18 Seconds	Das ist der eindrückliche Eisstrom des Aletschgletschers, der sich in der Schweiz befindet. Er ist der grösste und längste Gletscher in den Alpen. Doch seit 1850 ist er zurückgegangen. Grund dafür ist der menschengemachte Klimawandel. Die Situation ist alarmierend.<br><br>*This is the impressive ice stream of the Aletsch Glacier, located in Switzerland. It is the largest and longest glacier in the Alps. But since 1850 it has been receding. The reason for this is man-made climate change. The situation is alarming.*	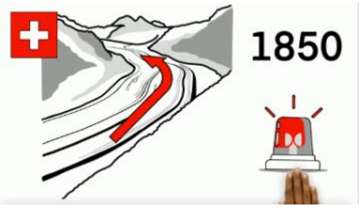
**Problem 1/4**<br><br>16 Seconds	Wir alle tragen mit unserem CO_2-Ausstoss dazu bei. Durchs Autofahren, Fliegen und Heizen werden Treibstoffe verbrannt und stossen so schädliche Gase aus. Diese gelangen dann in unsere Atmosphäre und verändern deren Zusammensetzung.<br><br>*We all contribute to this with our CO2 emissions. Driving, flying and heating burn fuels and emit harmful gases. These then enter our atmosphere and change its composition.*	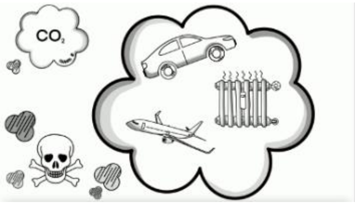
**Problem 2/4**<br><br>11 Seconds	Die Folge davon ist, dass mehr Sonnenstrahlen als bisher auf die Erde reflektiert werden. Wie im Treibhaus erwärmt sich dadurch die Luft auf der Erde deutlich mehr als normal.<br><br>*The result is that more of the sun's rays are reflected onto the earth than before. As in a greenhouse, this causes the air on Earth to heat up significantly more than normal.*	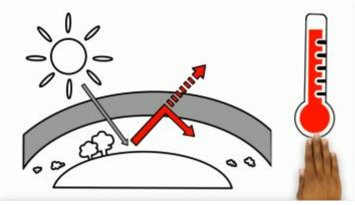

Problem 3/4

9 Seconds

Diese Erwärmung hat direkt spürbare Auswirkungen wie zum Beispiel Hitzewellen, Schmelzen der Gletscher oder heftige Regenfälle.

This warming has directly noticeable effects such as heat waves, melting of glaciers or heavy rainfall.

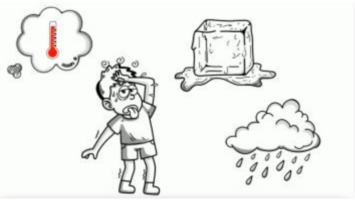

Problem 4/4

15 Seconds

Wir alle sind für diese extremen Ereignisse mitverantwortlich; denn im Durchschnitt verursachen wir pro Person 11 Tonnen CO_2 pro Jahr. Würden auf der ganzen Welt alle so verschwenderisch leben, bräuchten wir 3 Erden.

We are all partly responsible for these extreme events; because on average we cause 11 tons of CO2 per person per year. If everyone in the world lived so wastefully, we would need 3 earths.

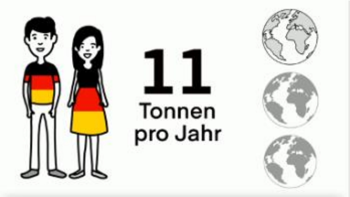

Solution 1/3

14 seconds

(ab 1:10)

Die gute Nachricht: Wir können unseren CO_2-Ausstoss kompensieren. Dies ist besonders sinnvoll bei Emissionen, die noch nicht vermeidbar sind. Zum Beispiel wenn du nicht auf einen Flug verzichten möchtest.

The good news is that we can offset our CO2 emissions. This is particularly useful for emissions that are not yet avoidable. For example, if you don't want to miss a flight.

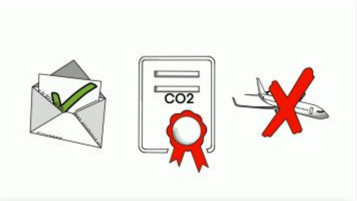

Manipulation 1

Dynamic / ISNA:

14 Seconds

Bei solchen Flugkompensationen zeigt sich ein starker Trend: Die Mehrheit befürwortet Kompensationen; und immer mehr Menschen kompensieren deshalb ihre Flugreisen.

There is a strong trend in such flight offsets: the majority is in favor of offsets; and more and more people are therefore offsetting their air travel.

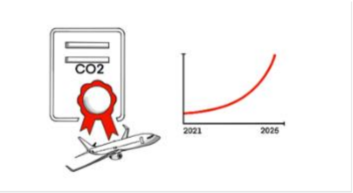

Dynamic / no ISNA:<br><br>10 Seconds	Bei solchen Flugkompensationen zeigt sich ein starker Trend: Immer mehr Menschen kompensieren ihre Flugreisen.<br><br>*A strong trend is emerging in such flight offsets: more and more people are offsetting their air travel.*	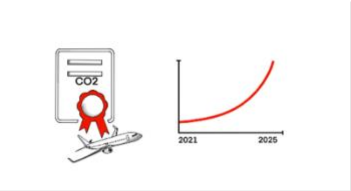
Static / ISNA<br><br>11 Seconds:	Obwohl die Mehrheit Kompensationen befürwortet, haben im Jahr 2022 allerdings erst sehr wenige Menschen ihre Flüge kompensiert.<br><br>*However, although the majority favors offsets, very few people have offset their flights in 2022.*	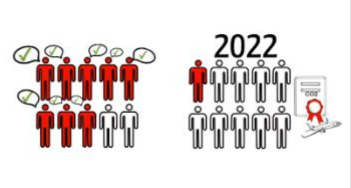
Static / no ISNA:<br><br>9 Seconds	Im Jahr 2022 haben allerdings erst sehr wenige Menschen ihre Flüge kompensiert.<br><br>*In 2022, however, only very few people have offset their flights.*	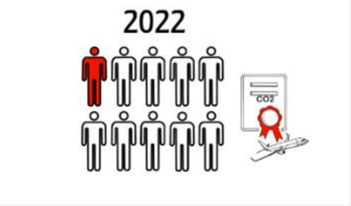
No DSNA / ISNA:<br><br>8 Seconds	Die meisten finden solche Kompensationen ein wichtiges Mittel zum Klimaschutz.<br><br>*Most people find such offsets an important means of climate protection.*	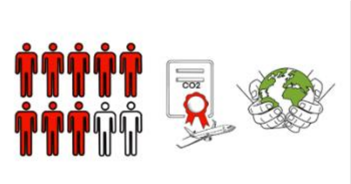
PNA:<br><br>8 seconds	Solche Kompensationen sind ein wichtiges Mittel zum Schutz des Klimas!<br><br>*Such offsets are an important means of protecting the climate!*	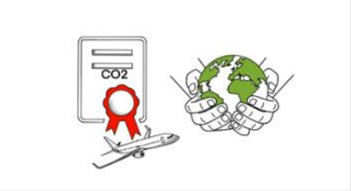
	**Constant in all videos**	
**Transition**<br><br>14 Seconds	Aber wie funktioniert die freiwillige Kompensation einer Flugreise?<br><br>Flugreisen können entweder direkt bei der Buchung der Reise kompensiert werden oder separat auf der Webseite eines Kompensationsanbieters.	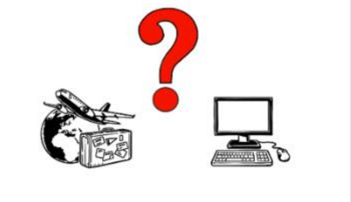

But how does voluntary offsetting of an air trip work?

Air travel can either be offset directly when booking the trip or separately on the website of an offset provider.

Solution 2/3

12 Seconds

Dafür muss man nicht immer tief in die Tasche greifen. Du kannst deinen Flug von Berlin nach Mallorca für 17 Euro oder einen Flug nach Bali für 100 Euro kompensieren.

You don't always have to dig deep into your pocket for this. You can offset your flight from Berlin to Mallorca for 17 euros or a flight to Bali for 100 euros.

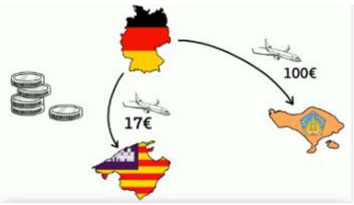

Solution 3/3

9 Seconds

Dieses Geld fliesst dann zum Beispiel in eine nachhaltige Waldbewirtschaftung in Deutschland oder in die Förderung von Solarenergie in Tansania.

This money then goes to sustainable forest management in Germany, for example, or to the promotion of solar energy in Tanzania.

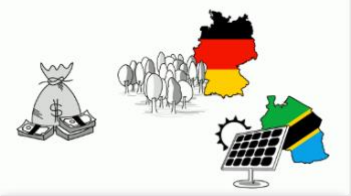

Explanation 1/2

9 seconds

Damit wird die durch den Flug ausgestossene Menge an CO2 an einer anderen Stelle auf der Erde eingespart und neutralisiert.

This means that the amount of CO2 emitted by the flight is saved and neutralized elsewhere on earth.

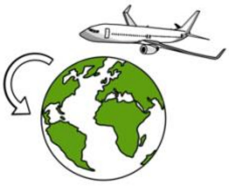

Explanation 2/2

17 seconds

Ärmere Länder profitieren dabei besonders, denn durch Klimaschutzprojekte wird auch Entwicklungsarbeit geleistet: CO2-Kompensationen helfen also dem Klima und gleichzeitig der lokalen Bevölkerung. Dadurch kann der menschengemachte Klimawandel verlangsamt werden.

Poorer countries benefit in particular, because climate protection projects also contribute to development work: CO2 offsets therefore help the climate and the local population at the same time. In this way, man-made climate change can be slowed down.

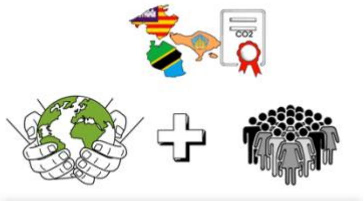

**Manipulation 2**		
**Static**<br>**DSNA**<br>14 Seconds	Doch wie viel wird in Deutschland kompensiert? Ein Blick auf die deutsche Bevölkerung zeigt, dass im Jahr 2021 nur eine von zehn Personen freiwillig ihren Flug kompensiert hat.<br><br>*But how much is compensated in Germany? A look at the German population shows that in 2021, only one in ten people voluntarily compensated for their flight.*	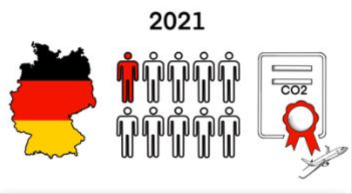
**Dynamic**<br>**DSNA**<br>20 Seconds	Doch wie viel wird in Deutschland kompensiert? Es zeigt sich ein starker Trend: *immer mehr* Menschen entscheiden sich für die Kompensation ihrer Flugreisen. Für die kommenden Jahre wird erwartet, dass sich dieser Trend weiter fortsetzen wird und deutlich mehr Flüge kompensiert werden als bisher.<br><br>*But how much is offset in Germany? A strong trend is emerging: more and more people are choosing to offset their air travel. This trend is expected to continue in the coming years, with significantly more flights being offset than before.*	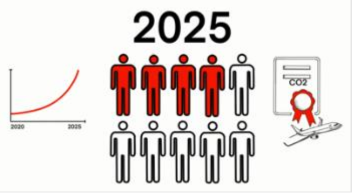
**ISNA**<br>20 seconds	Gleichzeitig zeigen viele Umfragen, dass eine deutliche Mehrheit der Deutschen Kompensationen befürwortet: 80% geben an, dass freiwillige CO2-Kompensationen im Flugverkehr sinnvoll, gut und wichtig sind.<br><br>*At the same time, many surveys show that a clear majority of Germans are in favor of offsets: 80% say that voluntary CO2 offsets in air travel are useful, good and important.*	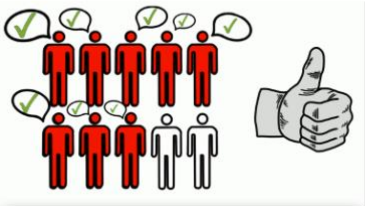
**ISNA (when there is no DSNA)**<br>20 seconds	Doch was halten die Deutschen von Kompensationen? Viele Umfragen zeigen, dass eine deutliche Mehrheit Kompensationen befürwortet: 80% geben an, dass freiwillige CO2-Kompensationen im Flugverkehr sinnvoll, gut und wichtig sind.	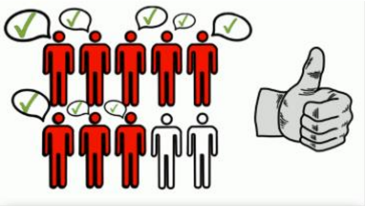

	But what do Germans think of offsets? Many surveys show that a clear majority is in favor of offsets: 80% say that voluntary CO2 offsets in air travel are sensible, good and important.	
**PN**<br><br>16 Seconds	Wir müssen unser Bestes geben. Wenn wir alle einen kleinen Teil beitragen, können wir den Klimawandel gemeinsam verlangsamen. Deine persönlichen Entscheidungen machen einen Unterschied – für dich und für andere.<br><br>*We have to do our best. If we all do a small part, we can slow climate change together. Your personal choices make a difference - for you and for others.*	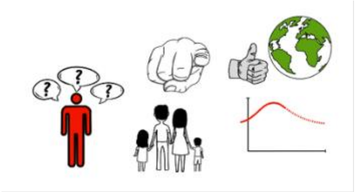
	**Konstant in allen Videos gleich**	
**End**<br><br>19 Seconds	Freiwillige CO_2-Kompensationen im Flugverkehr sind eine wichtige Lösung, um unseren Lebensraum und das Klima zu schützen. Gemeinsam sorgen wir so dafür, dass auch noch unsere Kinder die spektakulären Gletscher bestaunen können.<br><br>*Voluntary CO2 offsets for air travel are an important solution for protecting our habitat and the climate. Together, we can ensure that our children will still be able to marvel at the spectacular glaciers.*	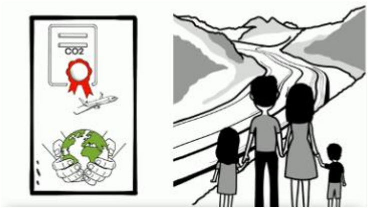

Note. The Storyboard is translated with AI (deepl.com). Explainer videos can be found here: https://osf.io/87y6t/?view_only=bb647eae503446d5b03aa58e777214fe

Measures

Variable	Statement (GER)
Subjective norms	Wie viele Ihrer Familienmitglieder, Freundinnen oder Freunde haben in der Vergangenheit schon eine Flugreise kompensiert? Schätzen Sie, wie groß der Anteil von denjenigen ist, die schon einmal eine Reise kompensiert haben. Bitte klicken Sie auf den Balken, um einen prozentualen Anteil festzulegen oder tragen Sie einen Wert in das Textfeld ein. Der Anteil liegt bei … Prozent.<br>(*How many of your family members, friends or acquaintances have already offset a flight in the past? Estimate the percentage of those who have already offset a trip. Please click on the bar to specify a percentage or enter a value in the text field. The share is ... percent*.)
Personal norms	Ich fühle mich moralisch dazu verpflichtet ... (*I feel morally obliged to...*)<br>… meine Flugreisen zu kompensieren. *(... to compensate for my air travel.*)<br>… mehr Geld für eine Flugreise auszugeben, wenn diese weniger klimaschädlich ist (z.B. Direktflug). *(... spend more money on a flight if it is less harmful to the climate (e.g. direct flight)*)<br>… mich über die Auswirkungen meiner Flugreise auf die Umwelt auseinanderzusetzen. *(... to think about the impact of my air travel on the environment*.)<br>… mich über alternative Transportmöglichkeiten neben dem Flugzeug zu informieren (z.B. Vergleich Zugreise). *(... to inform myself about alternative transport options besides air travel (e.g. comparison train travel)*)
Willingness to calculate emissions	Im Anschluss haben Sie die Möglichkeit, die Kosten für die Kompensation einer Flugreise zu berechnen. Möchten Sie den CO_2-Kompensationspreis für eine Ihrer Flugreisen berechnen?<br>(*In the following, you have the possibility to calculate the costs for offsetting an air flight. Would you like to calculate the CO2 offset price for one of your air travels?*)<br>• Ja, möchte ich berechnen. (*Yes, I would like to calculate.*)<br>• Nein, möchte ich nicht berechnen. (*No, I do not want to calculate*.)<br>• Ich bin in den letzten Jahren nicht geflogen. (*I have not flown in the last few years.*)
Willingness to pay for VCO	Für Ihre Flugreise von … Airport nach … Airport und zurück (… km, Economy Class) würde die Kompensation der Emissionen etwa … € Euro kosten. Wie wahrscheinlich ist es, dass Sie Ihre Flugreise zu diesem Preis kompensieren werden?<br>(*For your flight from [start] to [destination] ([distance] km, economy class), offsetting the emissions would cost about [price] Euro. How likely is it that you will offset your air travel at this price?*).

Note. The Items are translated with AI (deepl.com).
